# Mapping the temporality and structural impacts of modifications in *Escherichia coli* tRNAs

**DOI:** 10.1016/j.jbc.2026.111337

**Published:** 2026-03-03

**Authors:** Marcel-Joseph Yared, Carine Chagneau, Pierre Barraud

**Affiliations:** Expression génétique microbienne, Université Paris Cité, CNRS, Institut de biologie physico-chimique, IBPC, Paris, France

**Keywords:** transfer RNA, RNA modifications, modification circuit, pseudouridine, m^5^U, *Escherichia coli*, tRNA structure, TruB, TrmA, TrmB, NMR, kinetic assays

## Abstract

Transfer RNAs (tRNAs) are essential components of the protein synthesis machinery. Their biogenesis is a highly regulated process that involves the incorporation of numerous post-transcriptional chemical modifications, essential for tRNA folding, cellular stability and function. The sequential process by which these modifications are introduced remains poorly characterized. Previous studies have suggested the existence of modification hierarchies, particularly in the anticodon-loop region, but also among tRNA core modifications. Here, aiming to understand the molecular mechanisms by which modifications are incorporated in a bacterial model organism, we employed a combination of NMR spectroscopy and biochemical methods to characterize the maturation process of several *Escherichia coli* tRNAs. By monitoring tRNA maturation in a time-resolved fashion by NMR, we observed a conserved temporal pattern in the incorporation of the Ψ55, T54, and m^7^G46 modifications. We also show that Ψ55 stimulates the incorporation of T54 in *E. coli* tRNA^Phe^, tRNA^Val^, and tRNA^Asp^ and stimulates that of m^7^G46 in tRNA^Phe^ and tRNA^Asp^. Importantly, we also provide general insights into the impact of modifications on tRNA structural properties and show that while post-transcriptional modifications generally have a structuring effect that reduces conformational heterogeneities, these effects are tRNA-dependent, with certain tRNAs being more affected than others. These findings provide fundamental insights into the molecular aspects of tRNA maturation in *E. coli*.

Transfer RNAs (tRNAs) play a crucial role in the cellular process of protein synthesis and also have functions beyond translation (1, 2). To fulfil their diverse roles in the cell, tRNAs go through a meticulously regulated biogenesis process that results in mature tRNAs (3, 4, 5, 6). This biogenesis process generally involves the removal of the 5′-leader and 3′-trailer sequences from precursor tRNA transcripts, the addition of the 3′-CCA sequence for amino acid attachment, and the incorporation of numerous post-transcriptional chemical modifications. The ubiquitous presence of post-transcriptional chemical modifications in tRNAs is a hallmark of this RNA family, which exhibits the greatest diversity and number of modifications per molecule. Although about 100 different modifications have been identified in tRNAs, each tRNA typically has 5 to 15 modifications incorporated at specific sites along its sequence (7). These modifications are introduced in a controlled manner, ensuring the efficient formation of functional tRNAs (5, 8, 9). Many modified nucleotides are indeed crucial for the formation of tertiary interactions that create the L-shaped structure of tRNAs, which is essential for their stability and resistance to RNA degradation pathways (10, 11, 12, 13). Based on their location within the three-dimensional structure, modifications can be divided into two groups: those in the tRNA core and those in the anticodon-loop region (ACL). Modifications in the ACL mainly regulate translation fidelity and efficiency (14, 15), whereas those in the tRNA core mainly contribute to the folding and stability of tRNAs (3, 11). However, tRNA core modifications also play a role beyond tRNA stability in various cellular functions such as translation, cellular fitness, and stress adaptation, although the molecular mechanisms behind these functions are less well characterized than those involving ACL modifications (16).

Despite the critical role of tRNA modifications, our understanding of the multi-step process by which these modifications are introduced is still rather incomplete. Early mechanistic studies have shown that many tRNA-modifying enzymes can act on unmodified tRNAs transcribed *in vitro* (17). However, these completely unmodified tRNAs are likely not the enzymes preferred substrates. Indeed, several ‘modification circuits’ have been discovered, in which certain modifications either promote or inhibit the addition of subsequent modifications, suggesting a specific sequential order in the tRNA modification process (5, 18, 19). Most known examples of such ordered modification processes are found in the ACL region (18), and until recently, cross-talk involving modifications in the tRNA core were relatively rare (5, 20, 21). In recent years, with the aim of facilitating the discovery of modification circuits in tRNAs, we developed an NMR-based method to monitor the maturation of tRNAs in a time-resolved fashion (22). This technique revealed several modification circuits in the core of yeast tRNA^Phe^ (23). In particular, an important modification circuit was identified in the T-arm, where the presence of Ψ55 activates the formation of T54 (also referred to as m^5^U54) and m^1^A58, and T54 further promotes the formation of m^1^A58 (Positions and chemical structures of tRNA core modifications, and examples of modification circuits involving them, are summarized in Fig. S1) (23, 24).

In the bacterial model organism *Escherichia coli*, recent studies have provided insight into the interactions between tRNA core modifications. These reports indicate that the addition of acp^3^U47 is generally enhanced by the prior presence of m^7^G46 (25) and is also stimulated by T54 (26, 27). In addition, the incorporation of s^4^U8 is promoted by the presence of Ψ55 and T54 (27). Based on a collection of indirect evidence, an order for the introduction of modifications into *E. coli* tRNA^Phe^ was proposed. This order suggests that Ψ55 and T54 are among the first modifications introduced during tRNA maturation, followed by m^7^G46, acp^3^U47 and s^4^U8 (28). Furthermore, the enzyme TrmA, responsible for T54 formation, preferentially binds *E. coli* Ψ55-tRNA^Phe^ (28), suggesting that the Ψ55 → T54 branch present in numerous yeast elongator tRNAs (23, 29) may also regulate the T54 formation in other organisms, given that Ψ55 and T54 are highly conserved modifications across tRNAs and species (30).

To date, *E. coli* remains one of the few organisms where the complete set of tRNA modifications and the enzymes responsible for their introduction are known, making it one of the best-characterized tRNA maturation systems. However, the specific sequential steps of tRNA maturation and the interactions between different modifications during this process in *E. coli* have only recently begun to be systematically investigated. Indeed, a synthetic lethal screen recently identified epistatic relationships between tRNA modification genes in *E. coli*, revealing several pairs of modifications whose simultaneous deletion is either lethal or results in severe growth defects (31). Thus, interactions between modifications have been demonstrated at the genetic level, and gaining further insights in this area by investigating the temporality of introduction and the effect of modifications on modification enzyme activity would continue to deepen our overall understanding of tRNA maturation in bacteria.

Here, we employ a combination of NMR and biochemical methods to investigate the incorporation of modifications in *E. coli* tRNA^Phe^, tRNA^Val^, and tRNA^Asp^—selected as representative examples of *E. coli* tRNAs—which carry highly conserved modifications among *E. coli* tRNAs, namely Ψ55, T54, m^7^G46, and s^4^U8. First, by comparing imino (^1^H, ^15^N) correlation spectra of unmodified and modified sample of *E. coli* tRNAs, we provide insights into the tRNA-specific nature of the beneficial impact of modifications on the conformational stability of each tRNA. Next, through time-resolved NMR monitoring of tRNA maturation, we identified a conserved temporal pattern of modification incorporation in *E. coli* tRNAs. Interestingly, enzymatic activity assays combined with NMR monitoring of tRNA maturation reveal that catalytic efficiency of certain modification enzyme depends on the specific tRNA type. Finally, using time-resolved NMR monitoring of *E. coli* tRNA maturation in extracts of modification enzyme-deleted strains, we identify subtle interplays between modifications.

## Results

### Effect of modification incorporation on the structural properties of *E. coli* tRNA^Phe^, tRNA^Val^, and tRNA^Asp^

**Figure 1:**
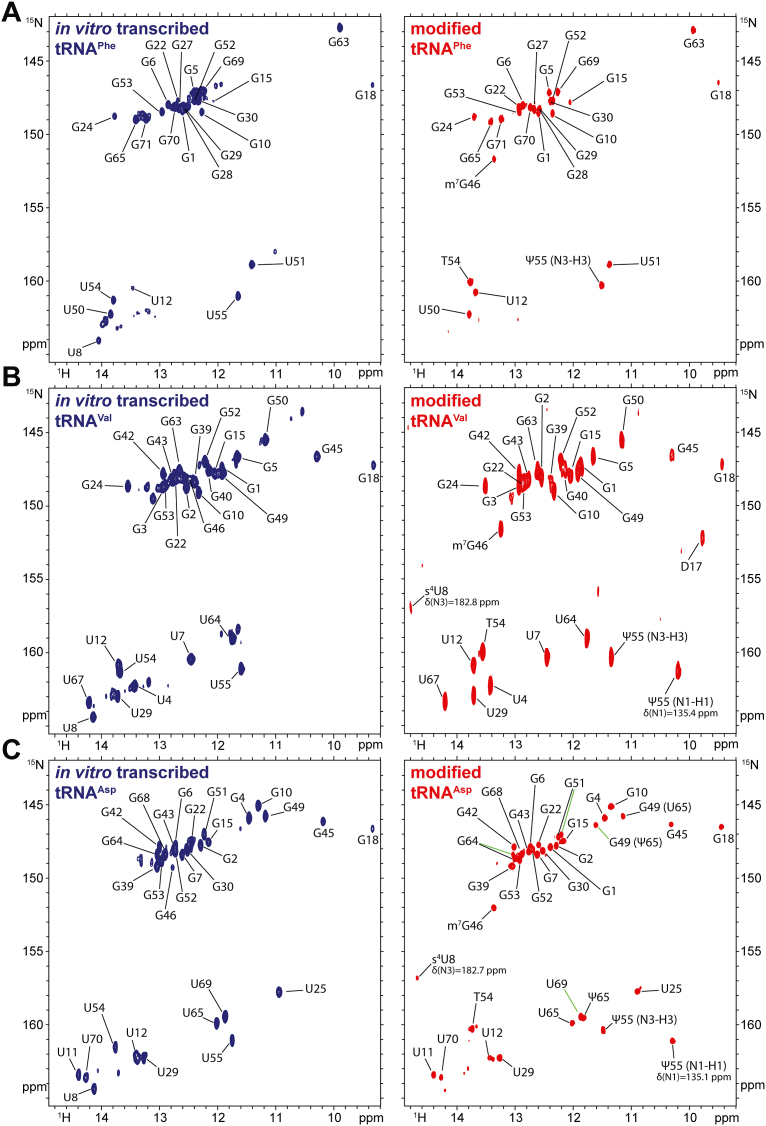
**Impact of modifications on the structural properties of *E. coli* tRNA^Phe^, tRNA^Val^, and tRNA^Asp^.** (^1^H,^15^N)-BEST-TROSY spectra and assignment of the imino groups of unmodified (in *blue*) and modified (in *red*) *E. coli* (*A*) tRNA^Phe^, (*B*) tRNA^Val^, and (*C*) tRNA^Asp^. Spectra were not all measured with the same number of points in the indirect ^15^N dimension. Details of NMR data measurement and processing have been reported in reference (32).

To evaluate the impact of post-transcriptional modifications on the structural properties of *E. coli* tRNA^Phe^, tRNA^Val^, and tRNA^Asp^, we measured ^1^H-^15^N correlation NMR spectra, in the imino region, on ^15^N-labeled samples of both unmodified and modified tRNAs. The unmodified transcripts were produced using standard *in vitro* transcription, while the modified samples were overexpressed and purified from *E. coli*. We next performed the chemical shift assignments of the imino groups of the unmodified and modified tRNAs (Fig. 1, *A*–*C*). The procedures followed, including those for sample preparation, and the extent of the NMR chemical shift assignment have been presented in detail in a previous technical report (32). In addition, Fig. S2 schematically summarizes the assigned resonances for each sample. Resonances corresponding to guanines and uridines involved in secondary or tertiary base pairing were successfully assigned in the unmodified and modified samples, such as those forming the acceptor, anticodon, D- and T-stems, as well as those involved in tertiary interaction, such as G18 and U55/Ψ55 (Figs. 1, *A*–*C* and S2). This indicates that tRNA^Phe^, tRNA^Val^, and tRNA^Asp^ globally adopt correct secondary and tertiary conformations, regardless of the modification status of these tRNAs.

However, while the global secondary and tertiary structures remain intact, the comparison of the NMR spectra for the unmodified and modified tRNAs revealed that the incorporation of modifications induces varying effects on the tRNA conformational equilibrium depending on the tRNA type (Fig. 1, *A*–*C*). For unmodified tRNA^Phe^, the spectra show signal broadening that leads to poorly defined NMR peaks, particularly in the guanine region, along with signal heterogeneity marked by a few low intensity peaks (U12, G15), and peaks with intermediate intensity that remained unassigned. These heterogeneities are absent in the spectra corresponding to modified tRNA^Phe^ (Fig. 1*A*). This shows that unmodified tRNA^Phe^ is heterogeneously folded into major and minor alternative conformations, which converge into a single major homogeneously folded conformation upon modifications incorporation. On the other hand, the spectra of unmodified tRNA^Val^ display some peaks with relatively high intensities that remain unassigned, as they do not correspond to the expected secondary and tertiary structure of tRNA^Val^. These unassigned peaks are no longer observable in the spectra of the modified sample (Fig. 1*B*). This observation indicates that unmodified tRNA^Val^ exists as heterogeneous mixture of at least two major conformations. The incorporation of modifications changes the folding properties and leads to a single properly folded tRNA structure. Finally, we noticed that the NMR spectra of both unmodified and modified tRNA^Asp^ exhibit well-defined peaks with only minor signal heterogeneity in the unmodified sample (Fig. 1*C*). This demonstrates that the incorporation of modifications has only minimal effect on the structural properties of tRNA^Asp^, making the structural impact of modifications unique to each of the three tRNAs.

### The NMR signature of individual modifications

**Figure 2:**
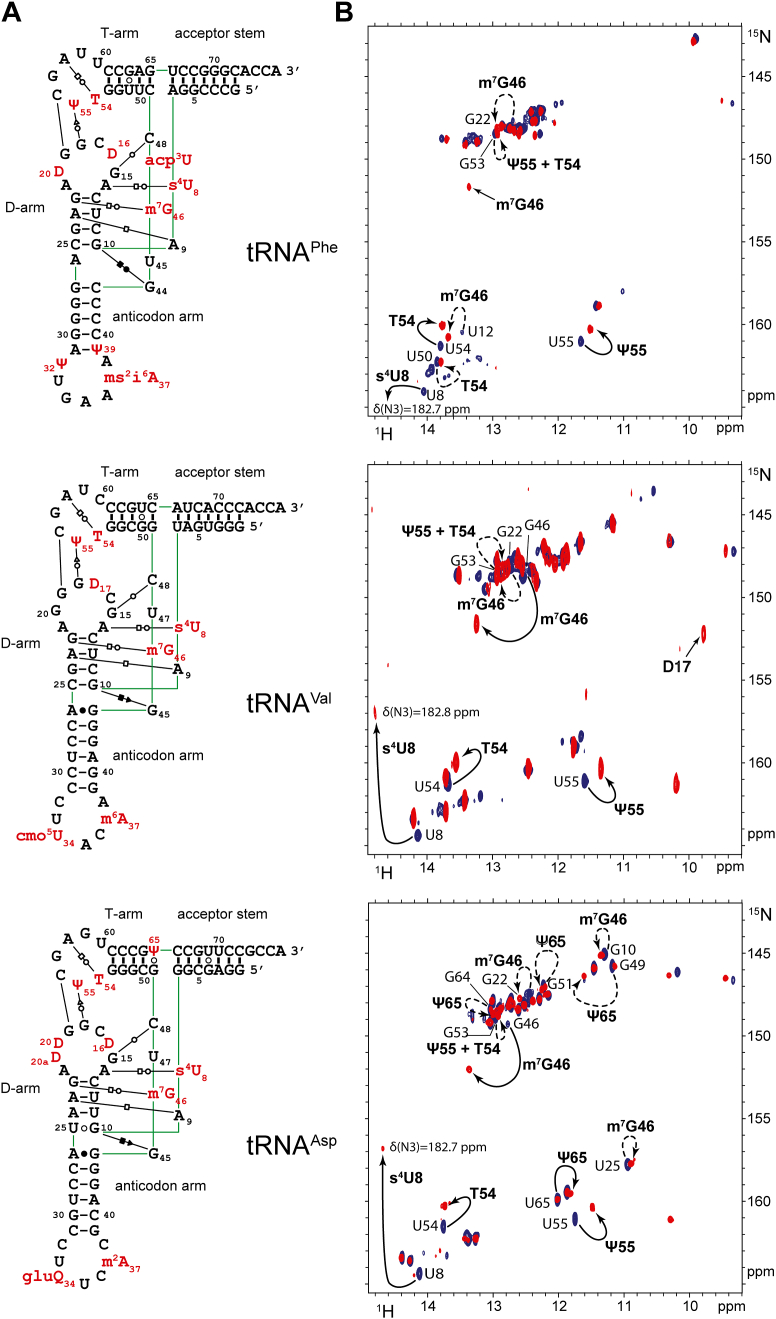
**The NMR signature of individual modifications in *E. coli* tRNA^Phe^, tRNA^Val^, and tRNA^Asp^.***A*, Sequence and L-shape 2D representation of modified *E. coli* tRNA^Phe^ (*top*), tRNA^Val^ (*middle*) and tRNA^Asp^ (*bottom*). The nature of interactions between base pairs is described according to the classification by Leontis and Westhof (49). *B*, superposition of (^1^H,^15^N)-BEST-TROSY spectra of unmodified (in *blue*) and modified (in *red*) *E. coli* tRNA^Phe^ (*top*), tRNA^Val^ (*middle*) and tRNA^Asp^ (*bottom*), revealing the NMR signature of individual modifications. NMR data measurement and processing have been reported in reference (32). A selection of imino assignments is reported on the spectra. NMR signatures of modifications are reported with continuous line arrows (direct effects), or dashed arrows (indirect effects).

The incorporation of modifications affects the chemical environment of both the modified nucleotides as well as their neighbors, leading to shifts in their corresponding NMR peaks. To characterize the impact of modifications on the NMR fingerprints of tRNA^Phe^, tRNA^Val^, and tRNA^Asp^, we compared the NMR spectra of the modified and unmodified tRNAs, revealing the NMR signature of individual modifications (Fig. 2). Note that below and in the entire manuscript, we use ‘direct effect’ to refer to a chemical shift change caused by a modification on the nucleotide itself, and ‘indirect effect’ for changes caused by a modification on a neighboring nucleotide. By analyzing these overlays in the context of the known secondary and tertiary structures of these tRNAs (Fig. 2*A*), we identified both direct and indirect effects, depicted on Figure 2*B* with plain and dashed lines, respectively. Specifically, we identified the NMR signature of Ψ55, T54, m^7^G46, and s^4^U8 in tRNA^Phe^, tRNA^Val^, and tRNA^Asp^ as well as D17 in tRNA^Val^ and Ψ65 in tRNA^Asp^. For instance, in the case of T54, we observed a direct effect on the chemical shift of the peak of U54 and an indirect effect on the chemical shift of the peak corresponding to the neighboring G53 (Fig. 2*B*).

However, NMR signatures were not identified for all modifications. For instance, modifications in the ACL are not easily detectable by NMR, since they belong to single-stranded regions where imino groups are unprotected from exchange with the solvent. Similarly, dihydrouridines in the D-loop are not always detectable. As a result, their NMR signature must be inferred from variations in the chemical shifts of neighboring residues (indirect effects). This complicates the unambiguous identification of the NMR signature of modifications in an unpaired context. Nevertheless, the NMR signatures that we identified (see above) allowed us to monitor the introduction of modifications in the three tRNAs in a time-resolved fashion using NMR.

### Time-resolved monitoring of modification incorporation in tRNA^Phe^, tRNA^Val^, and tRNA^Asp^

**Figure 3:**
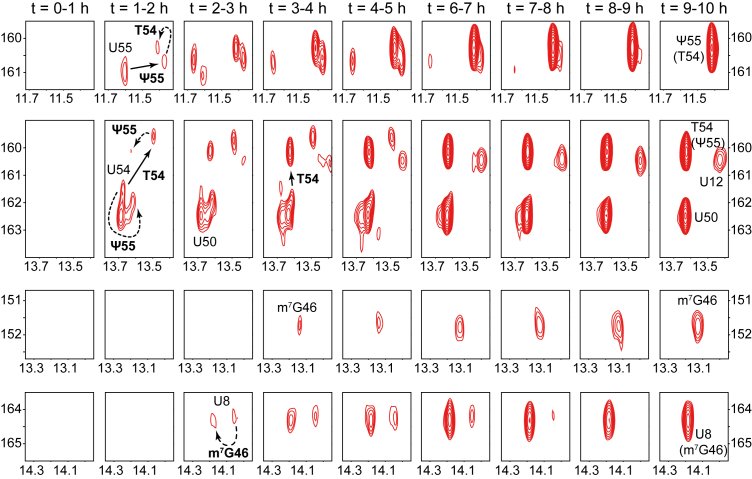
**Time-resolved NMR monitoring of tRNA^Phe^ maturation.** Selected imino (^1^H,^15^N) correlation spectral regions extracted from time-resolved NMR spectra of ^15^N-labeled tRNA^Phe^ during continuous incubation in wild-type *E. coli* extract at 30 °C (see Fig. S3). Each NMR spectrum measurement spreads over a 1 h time period, as indicated. Each panel (*row*) focuses on a specific spectral region to monitor the time-dependent evolution of specific modifications. Detected modifications are reported with continuous line arrows for direct effects, or dashed arrows for indirect effects. Ψ55 and T54 are both monitored in the top two panels, either directly (*via* U55 and U54, respectively) or indirectly (*via* T54 and Ψ55, respectively). m^7^G46 is monitored both directly in the third panel and indirectly *via* U8 in the *bottom panel*. The position of a signal *in the presence* of a specific modification is noted with the specific modification inside brackets under the nucleotide label.

**Figure 4:**
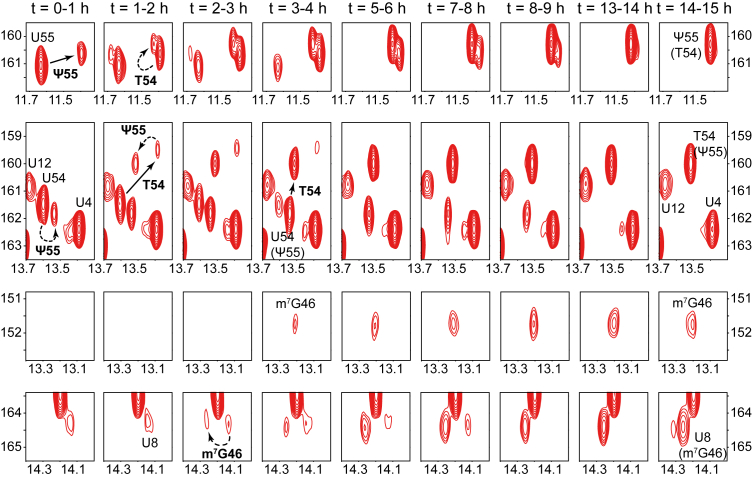
**Time-resolved NMR monitoring of tRNA^Val^ maturation.** Selected imino (^1^H,^15^N) correlation spectral regions extracted from time-resolved NMR spectra of ^15^N-labeled tRNA^Val^ during continuous incubation in wild-type *E. coli* extract at 30 °C (see Fig. S4). Each NMR spectrum measurement spreads over a 1 h time period, as indicated. Each panel (*row*) focuses on a specific spectral region to monitor the time-dependent evolution of specific modifications. Detected modifications are reported with continuous line arrows for direct effects, or dashed arrows for indirect effects. Ψ55 and T54 are both monitored in the top two panels, either directly (*via* U55 and U54, respectively) or indirectly (*via* T54 and Ψ55, respectively). m^7^G46 is monitored both directly in the third panel and indirectly *via* U8 in the *bottom panel*. The position of a signal *in the presence* of a specific modification is noted with the specific modification inside brackets under the nucleotide label.

**Figure 5:**
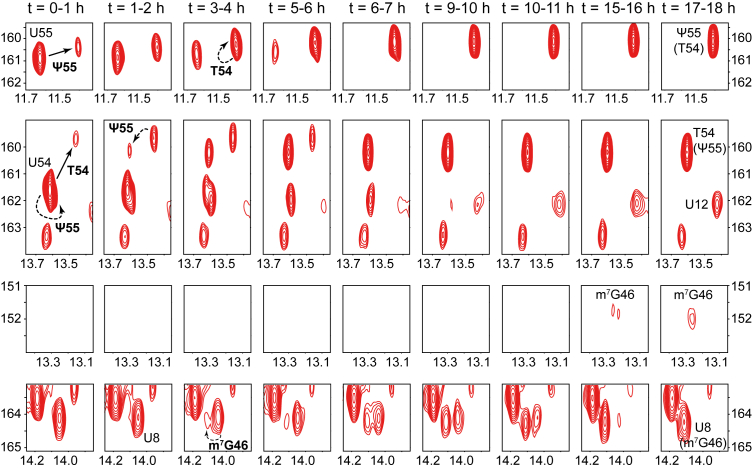
**Time-resolved NMR monitoring of tRNA^Asp^ maturation.** Selected imino (^1^H,^15^N) correlation spectral regions extracted from time-resolved NMR spectra of ^15^N-labeled tRNA^Asp^ during a continuous incubation in wild-type *E. coli* extract at 30 °C (see Fig. S5). Each NMR spectrum measurement spreads over a 1 h time period, as indicated. Each panel (*row*) focuses on a specific spectral region to monitor the time-dependent evolution of specific modifications. Detected modifications are reported with continuous line arrows for direct effects, or dashed arrows for indirect effects. Ψ55 and T54 are both monitored in the top two panels, either directly (*via* U55 and U54, respectively) or indirectly (*via* T54 and Ψ55, respectively). m^7^G46 is monitored both directly in the third panel and indirectly *via* U8 in the *bottom panel*. The position of a signal *in presence* of a specific modification is noted with the specific modification inside brackets under the nucleotide label.

In order to monitor the incorporation of modifications using NMR, we incubated each of the unmodified ^15^N-labeled transcripts of tRNA^Phe^, tRNA^Val^, and tRNA^Asp^ in unlabeled *E. coli* cell extracts supplemented with enzymatic cofactors involved in the introduction of modifications, namely S-adenosyl-L-methionine (SAM) and nicotinamide adenine dinucleotide phosphate (NADPH). We then measured a series of ^1^H-^15^N BEST-TROSY experiments, each having an acquisition time of 1 h, starting from the initial incubation and extending to approximately 24 h. We adapted our previously published methodology, originally developed for yeast extracts (22, 23), to *E. coli* bacterial extracts. We diluted the bacterial extract by a factor 3 to globally slow-down the modification process (see below and experimental procedures). Each NMR spectrum provides a snapshot of all modification events that occurred during the 1-h acquisition period. We identified these modification events by linking changes in the NMR fingerprint to the NMR signatures of individual modifications (Fig. 2). This series of spectra thus allowed us to monitor the incorporation of modifications over time. Data are presented as small spectral regions (Figure 3, Figure 4, Figure 5), alongside the corresponding full imino spectra (Figs. S3–S5). Together, these representations illustrate the evolution of the modified species and facilitate comparison among the different modifications. The absence of identifiable NMR signatures for dihydrouridines and acp^3^U47 prevented us from detecting their incorporation, and their introduction during the incubation experiment thus remains uncertain. In addition, although distinct and well-defined NMR signatures were identified for s^4^U8 incorporation in all three tRNAs and Ψ65 incorporation in tRNA^Asp^—specifically, a downfield shift of the U8 N3 resonance by approximately +20 ppm (Fig. 2) and substantial chemical shift changes in the U65 N3-H3 and G49 N1-H1 signals upon conversion to Ψ65 (Fig. 2*C*)—these characteristic changes were not observed during the incubation experiments (Figure 3, Figure 4, Figure 5). This indicates that s^4^U8 and Ψ65 were not introduced into the tRNAs incubated in cell extracts. For s^4^U8, this is most probably due to the inability of our extract to recapitulate the thiolation reaction, despite extensive optimization attempts (data not shown). Although the formation of the s^4^U8 modification has been already achieved *in vitro* with purified enzymes (28, 33), thiolation reactions are generally challenging in cellular extracts due to their sensitivity to oxidation. Importantly, the observation of Ψ55, T54, and m^7^G46 incorporation was unambiguous in all three tRNAs (Figure 3, Figure 4, Figure 5). We observed that these modifications were introduced with similar timing. Importantly, we consider the modification to be fully incorporated once the signal associated with its NMR signature reaches a plateau and remains stable across successive spectra. Overall, Ψ55 and T54 are introduced almost in the same timeframe, with m^7^G46 following shortly after (Figure 3, Figure 4, Figure 5 and Table 1). In the case of tRNA^Phe^ and tRNA^Asp^, our experimental setup did not allow us to detect a distinct introduction of Ψ55 and T54, since their initial appearance occurs in the same spectra (Figs. 3 and 5). In the case of tRNA^Val^, we could observe a separate incorporation of Ψ55, T54, and m^7^G46 over time (Fig. 4). Notably, it was necessary to dilute the cell extracts in order to capture the introduction of Ψ55, T54, and m^7^G46 at distinct time points, which we did for all three tRNA maturation monitoring. Without dilution, the enzymatic activities in the extracts were leading to the rapid completion of all modifications within the first acquired spectrum (data not shown).

**Table 1:** Time-resolved NMR monitoring of modification incorporation in tRNA^Phe^, tRNA^Val^, and tRNA^Asp^

tRNA	Modification	Modification [onset - completion] time^a^	Duration of incorporation
tRNA^Phe^	Ψ55	1–8 h	7 h
	T54	1–9 h	8 h
	m^7^G46	2–9 h	7 h
tRNA^Val^	Ψ55	0–5 h	5 h
	T54	1–14 h	13 h
	m^7^G46	2–9 h	7 h
tRNA^Asp^	Ψ55	0–6 h	6 h
	T54	0–10 h	10 h
	m^7^G46	2–18 h	16 h

a As seen in the NMR maturation experiments (see Figure 3, Figure 4, Figure 5).

Importantly, although the initiation of the introduction of Ψ55, T54, and m^7^G46 was detected at similar time points in tRNA^Phe^, tRNA^Val^, and tRNA^Asp^, we noticed that the duration required to complete some of these modifications varied depending on the tRNA (Figure 3, Figure 4, Figure 5 and Table 1). This suggests that the introduction of modifications, reflecting the activity of the modification enzymes, depends on the substrate identity, especially for m^7^G46 (see below). In detail, the NMR monitoring of *E. coli* tRNA^Phe^ maturation reveals that the introduction of Ψ55 starts after approximately 1 h of incubation and is completed after ∼8 h (*i.e.* lasting 7 h), that T54 is incorporated between ∼1 to 9 h (*i.e.* lasting 8 h), while m^7^G46 is introduced between ∼2 to 9 h (*i.e.* lasting 7 h) (Fig. 3 and Table 1). In the monitoring of the maturation of tRNA^Val^, we noticed that the introduction of Ψ55 is detected immediately after incubation (t = 0–1 h) and is fully incorporated after ∼ 5 h of incubation (a 5-h duration) (Fig. 4 and Table 1). We also observed that T54 is incorporated over a 13-h period, between ∼1 and 14 h, while m^7^G46 is introduced between ∼2 to 9 h (*i.e.* lasting 7 h) (Fig. 4 and Table 1). For tRNA^Asp^, the timing of modification incorporation indicates that Ψ55 and T54 start being introduced directly after incubation (t = 0–1 h), with Ψ55 completing after ∼6 h (a 6-h duration) and T54 after ∼10 h (a 10-h duration) (Fig. 5 and Table 1). Meanwhile, m^7^G46 is introduced between ∼2 to 18 h, highlighting a notably long 16-h duration for m^7^G46 modification in tRNA^Asp^ (Fig. 5 and Table 1).

Thus, the kinetic parameters for the introduction of Ψ55 and T54 in tRNA^Phe^, tRNA^Val^, and tRNA^Asp^ are relatively similar. However, m^7^G46 formation differs, requiring significantly more time to be incorporated in tRNA^Asp^. This indicates that while TruB and TrmA seem to act similarly on tRNA^Phe^, tRNA^Val^, and tRNA^Asp^ transcripts, tRNA^Asp^ is likely a poor substrate for m^7^G46 formation by TrmB.

### tRNA^Asp^ is a poor substrate for m^7^G46 formation compared to tRNA^Phe^ and tRNA^Val^

**Figure 6:**
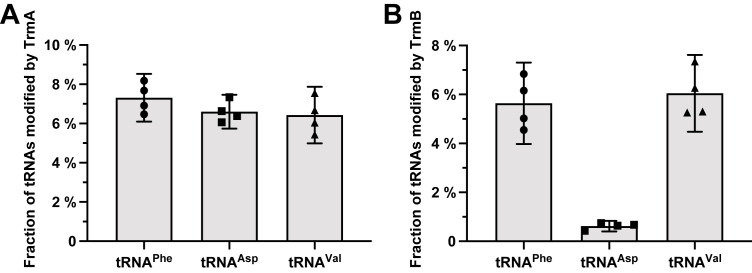
**Influence of the tRNA substrate on the enzymatic activity of TrmA and TrmB.***A and B*, bar chart comparing the fractional conversion of the substrate, *i.e.* the fraction of T54 introduced by TrmA (*A*), and m^7^G46 introduced by TrmB (*B*) in tRNA^Phe^, tRNA^Asp^ and tRNA^Val^ (in %). Names of the tRNAs are indicated below the graph. Black dots represent individual measurements. Modified tRNA quantities were measured for 1 time point at t = 10 min in four independent experiments (N = 4). Data heights represent the mean of the replicates. Error bars correspond to the confidence interval at 95% (CI 95%). Reactions remained in the initial linear phase of product formation under these conditions (Fig. S6*A*).

To better characterize the modification process of tRNA^Phe^, tRNA^Val^, and tRNA^Asp^ and to identify good and poor substrates for T54 and m^7^G46 formation, we conducted enzymatic activity assays for TrmA and TrmB on the unmodified transcripts of these tRNAs. We observed no marked difference in the introduction of T54 (Figs. 6 and S6), which shows that TrmA act similarly on these three tRNAs. In contrast, we observe that the initial rate of m^7^G46 incorporation by TrmB is much lower for unmodified tRNA^Asp^ than for unmodified tRNA^Phe^ and tRNA^Val^ (Figs. 6 and S6). This is in line with the difference observed in NMR, *i.e.* a 10-h difference for tRNA^Asp^ compared to tRNA^Phe^ and tRNA^Val^. Altogether, this shows that tRNA^Asp^ is a poor substrate for m^7^G46 formation by TrmB compared to tRNA^Phe^ and tRNA^Val^.

### Impact of modifications on the maturation of *E. coli* tRNA^Phe^, tRNA^Val^, and tRNA^Asp^

**Figure 7:**
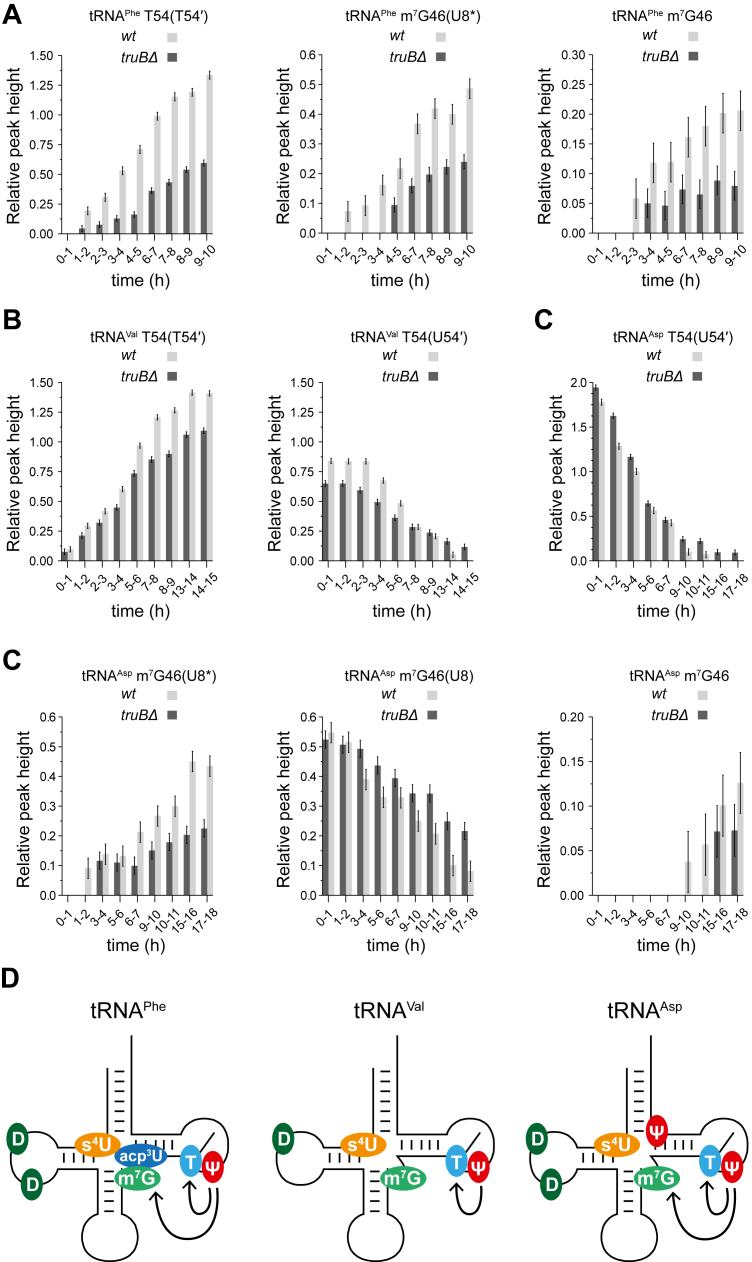
**Impact of modifications on the maturation of *E. coli* tRNA^Phe^, tRNA^Val^, and tRNA^Asp^.***A*, Bar chart showing the time-resolved evolution of the relative intensity of NMR signals reporting on T54 and m^7^G46 formation in tRNA^Phe^, monitored in wild-type (*wt*) and *truBΔ* extracts. Here, the combined signal T54′ reflects the total T54-related signal when U55 exists in different modification states in the tRNA population. The relative peak height value for T54′ includes the contribution of the T54 peak (tRNA with unmodified U55) and the shifted T54∗ peak (tRNA with modified Ψ55). Error bars represent the reported uncertainty in peak heights, calculated as ±2 × the normalized mean RMS of the noise, thereby approximating a 95% confidence interval (CI 95%; see Experimental Procedures). *B*, time-resolved evolution of the relative peak height of NMR signals reporting on T54 formation in tRNA^Val^, monitored in *wt* and *truBΔ* extracts. Here, the relative peak height value for T54′/U54′ includes the contribution of the T54/U54 peak (tRNA with unmodified U55) and the shifted T54∗/U54∗ peak (tRNA with modified Ψ55). *C*, time-resolved evolution of the relative peak height of NMR signals reporting on T54 and m^7^G46 formation in tRNA^Asp^, monitored in *wt* and *truBΔ* cell extracts. *D*, schematic view of the positive influences revealed by the NMR monitoring of tRNA^Phe^, tRNA^Val^, and tRNA^Asp^ maturation in *E. coli* extracts.

The timing differences observed in the incorporation of modifications in these tRNAs may reflect that the modifications influence each other. Aiming to investigate how modifications affect one another in *E. coli* tRNAs, we measured multiple time-resolved NMR spectra of tRNA^Phe^, tRNA^Val^, and tRNA^Asp^ modification pathways in different extracts prepared from *E. coli* strains deleted each of a specific modification enzyme (Figs. S7–S25). The resulting series of snapshots describing the modification patterns were then compared to those observed in the wild-type *E. coli* extract, revealing subtle interplays between modifications. We first performed a qualitative visual comparison of the different NMR spectra, noting that the differences are most apparent when examining the full series of maturation spectra (Figs. S7–S25). We then confirmed these observations through a quantitative analysis of the relative NMR peak height over time for modification-specific signals reporting on the incorporation of T54 and m^7^G46 (Figs. 7, *A*–*C* and S26). Depending on the tRNA examined and given inherent differences in how reliably specific signals report modification incorporation, multiple probes were analyzed. For T54, we quantified either the increase in intensity of the T54 imino signal (Fig. 7, *A* and *B*) or the decrease in intensity of the U54 imino signal (Fig. 7, *B* and *C*). For m^7^G46, we quantified either the decrease in the U8 imino signal, the increase in the shifted U8 imino signal (U8∗), or the increase in the m^7^G46 imino signal (Fig. 7, *A*–*C*).

For tRNA^Phe^, we notice a slight delay in the incorporation of T54 and m^7^G46 modification in the *truBΔ* strain lacking Ψ55 (Fig. 7*A*). This shows that the prior presence of Ψ55 may positively influence the incorporation of T54 and m^7^G46 on *E. coli* tRNA^Phe^ (Fig. 7*D*). Similarly, for tRNA^Asp^, we observe a delay in the incorporation of T54 and m^7^G46 modification in the *truBΔ* strain lacking Ψ55 (Fig. 7*C*), showing that Ψ55 positively impacts the incorporation of T54 and m^7^G46 on *E. coli* tRNA^Asp^ (Fig. 7*D*). Finally, for tRNA^Val^, we observe a delay in the incorporation of T54 in the *truBΔ* strain lacking Ψ55 (Fig. 7*B*), showing that the introduction of T54 is positively influenced by the prior presence of Ψ55 (Fig. 7*D*). Thus, even though the influence of modifications may generally be substrate-dependent, the stimulation of T54 incorporation in the presence of Ψ55 is conserved in the 3 *E. coli* tRNAs studied here. Importantly, although the observed delay in the incorporation of T54 and m^7^G46 modifications can be substantial (few hours), the actual effects might be more subtle considering the dilution of the extracts used here. In conclusion, the introduction of modifications in tRNA^Phe^, tRNA^Val^ and tRNA^Asp^ is not strictly dependent on the presence of prior modifications, but only slightly affected by initial ones.

## Discussion

In this study, NMR and biochemical approaches were used in conjunction to study the temporality of modification incorporation in *E. coli* tRNAs. In this approach, NMR is particularly beneficial to provide structural information on the effect of modifications on tRNA conformations. The interconnections between the temporal pattern of modification incorporation, the efficiency of modification introduction depending on the tRNA type, and the conformational heterogeneity in the presence or absence of modifications are discussed below.

By monitoring the introduction of modifications by time-resolved NMR, we observed a defined timing of modification incorporation in *E. coli* tRNA^Phe^, tRNA^Val^ and tRNA^Asp^, in which Ψ55 and T54 appear first, followed by m^7^G46. This timing pattern is consistent with a previously proposed order of modification introduction in *E. coli* tRNA^Phe^ (28). That study shows that TruB preferentially binds and modifies unmodified tRNA^Phe,^ and TrmA preferentially binds to Ψ55-tRNA^Phe^ (28). In addition, the presence of m^7^G46 inhibits the introduction of Ψ55 by TruB and T54 by TrmA, while the presence of Ψ55 and T54 does not affect the incorporation of m^7^G46 by TrmB (28). Based on these binding and kinetic parameters, the authors suggested that Ψ55 and T54 are introduced in the early steps of maturation, while m^7^G46 is formed later. Here, using time-resolved NMR, we observed the introduction of Ψ55, T54 and m^7^G46 with a specific timing that is consistent with the previously proposed order. Notably, a similar temporal pattern for the incorporation of Ψ55, T54 and m^7^G46 is also found in yeast tRNA^Phe^ (23), suggesting that the introduction of these modifications in the early steps of maturation is likely a widespread feature of tRNA maturation.

With time-resolved NMR and activity assays conducted for TrmA and TrmB, we demonstrate that T54 is incorporated similarly across all three tRNAs studied here, with only minor variations in the catalytic efficiency of TrmA. However, we show that the incorporation of m^7^G46 by TrmB is substantially less efficient in tRNA^Asp^ compared to its formation in tRNA^Phe^ and tRNA^Val^ (Fig. 6). Similarly, we previously showed that in yeast, unmodified tRNA^Phe^ is a poor substrate for Trm6/Trm61, which catalyzes m^1^A58 formation, whereas unmodified initiator tRNA_i_^Met^ is a good substrate for the same enzyme (24). Notably, m^1^A58 is essential for the proper folding of initiator tRNA_i_^Met^ into a homogeneous structure (24). In this context, it would be interesting to investigate the effect of m^7^G46 on the conformational stability of both good and poor substrates of TrmB, exploring a potential link between these two aspects.

We also identified slight positive influences between modifications in *E. coli* tRNAs. Notably, the interplay identified in *E. coli* tRNA^Phe^, tRNA^Val^ and tRNA^Asp^ between Ψ55 and T54 is also present in several yeast tRNAs (23, 29). The observed impacts of modifications described in this study differ in some respects from those reported previously (28). Although the authors demonstrated a preferential binding of TrmA on *E. coli* Ψ55-tRNA^Phe^, which is consistent with the Ψ55 → T54 positive influence identified here, they did not observe any increase in the *in vitro* enzymatic activity of TrmA in the presence of Ψ55 on *E. coli* tRNA^Phe^. This difference may stem from the conditions under which the enzymatic experiments are conducted. In the complex environment of cellular extracts, interactions with different enzymes, the tRNAs, and/or other factors may influence the activity of individual enzymes, potentially contributing to the observed differences.

In the present study, we show that *E. coli* tRNA^Phe^, tRNA^Val^, and tRNA^Asp^ each display distinct conformational behaviors in solution when unmodified. Specifically, unmodified tRNA^Phe^ exhibits a high level of conformational heterogeneity in solution, unmodified tRNA^Val^ exists as a heterogeneous mixture of at least two major conformations, and unmodified tRNA^Asp^ adopts a relatively homogeneous conformation in solution (Fig. 1 and paragraph ‘Effect of modification incorporation on the structural properties of *E. coli* tRNA^Phe^, tRNA^Val^, and tRNA^Asp^’). These structural differences appear to be specific to each tRNA, as previously observed in other examples (24, 34, 35, 36). Recently, the description of large conformational dynamics in tRNAs have been reported (37, 38). These studies, based on ^15^N relaxation and high-pressure NMR, suggest that conformational reorganization in tRNA could be linked to maturation and folding (37, 38). Combined with our observation that conformational heterogeneity varies between tRNAs, this highlights the need for detailed studies on tRNA conformational dynamics across multiple tRNA systems to better characterize the range of existing structural behaviors. Furthermore, the benefits conferred by specific modifications also appear to be tRNA-specific. As seen in this study, modifications appear to stabilize the structure of tRNA^Phe^ and tRNA^Val^ but seem to have little to no effect on tRNA^Asp^. This finding aligns with a previous report based on molecular dynamics simulations, which indicated that modifications have distinct structural effects depending on the tRNA (39). It also supports the idea that not all modifications are beneficial for all tRNAs (40). For instance, the absence of m^7^G46 has been linked to the specific degradation of *Saccharomyces cerevisiae* tRNA^Val^ (12, 41, 42), *S. pombe* tRNA^Tyr^ and tRNA^Pro^ (43) and *Thermus thermophilus* tRNA^Phe^ and tRNA^Ile^ (20). Interestingly, m^1^A58 plays a major role in yeast initiator tRNA_i_^Met^, where it is responsible for proper tertiary folding and for the protection against degradation, while having no major effect on other yeast tRNAs (24, 44, 45, 46). Since the structural behavior, the catalytic efficiency of modification enzymes, and the benefits of particular modifications are all tRNA-specific—and, in some cases, interconnected (see above)—it is reasonable to propose that the varying degrees of conformational heterogeneity observed in the different *E. coli* tRNAs represent a fundamental aspect of tRNA structural properties. This trait is likely a key factor underlying the differences in how individual tRNAs are sensitive to degradation in the absence of certain modifications.

## Experimental procedures

### *E. coli* strains

*E. coli* strains used in this study are listed in Table S1. The wild-type *E. coli* BW25113 strain, and the strains carrying deletions of the genes for modification enzymes (ECK2955, ECK4041, ECK2133, ECK3957, ECK3155, ECK2581, ECK0417, ECK3247, ECK2785) were obtained from the kanamycin-resistant *E. coli* Keio collection (47), and were used to prepare cell extracts. Deletion of the targeted gene and its replacement by the kanamycin resistance cassette were checked with PCR using appropriate sets of primers (Table S2). Correspondence between tRNA modifications and the enzymes responsible for their introduction in *E. coli* is reported in Table S3.

### *E. coli* extract preparation

The preparation of the cellular extracts was adapted from a previous procedure for yeast cell extracts (22). *E. coli* cells from the BW25113 Keio collection (WT and deleted strains) were grown at 37 °C in LB (Luria Bertani) medium until the OD_600_ stabilizes (*i.e.* 7 h) and harvested by centrifugation. Pellets were stored at −80 °C and subsequently thawed and resuspended in a volume of lysis buffer (Na_2_HPO_4_/KH_2_PO_4_ pH 7.0 25 mM, MgCl_2_ 10 mM, EDTA 0.1 mM) equivalent to 3 times the weight of the cell pellet (*i.e.* 3 ml of lysis buffer per g of cells). The dilution of the extract using this 3:1 volume-to-weight ratio was necessary to reduce the enzymatic activity of the extract (see main text). The mix is then complemented with 2 mM dithiothreitol (DTT), 1 mM phenylmethylsulphonyl fluoride (PMSF), 1 mM benzamidine, and 1 μg/ml each of leupeptin, pepstatin, antipain, and chymostatin. Cells were rapidly frozen to −80 °C in an Eaton pressure chamber and lysed at 30,000 psi in a hydraulic press. After unfreezing of the homogeneate, DTT, PMSF, benzamidine, leupeptin, pepstatin, antipain and chymostatin were added to double their initial concentration. The homogenate was then centrifuged at 30,000×*g* for 1 h at 8 °C to remove cellular debris. The supernatant was further centrifuged at 100,000×*g* for 1 h at 8 °C. The resulting supernatant was quickly aliquoted and frozen in liquid nitrogen. Aliquots were stored at −80 °C until further use.

### Modification enzyme cloning

Each of the genes encoding the full-length *E. coli* TruB (M1 to A314 – Uniprot entry P60340), the full-length *E. coli* TrmB (M1 to K239 – Uniprot entry P0A8I5) and the full-length *E. coli* TrmA (M1 to K366 – Uniprot entry P23003) were cloned from BW25113 genomic DNA between the *NheI* and *BamHI* sites of a pET28a vector (Novagen) using appropriate primers for PCR amplification (Table S2). All recombinant proteins were engineered with an N-terminal His-tag and a downstream thrombin cleavage site (MGSSHHHHHHSSGLVPRGSH) to enable efficient affinity purification and optional tag removal.

### Overexpression and purification of the modification enzymes

TruB, TrmB and TrmA were overexpressed in *E. coli* BL21(DE3) (Agilent) in LB media. The cells were grown at 37 °C and induced at OD_600_ ∼0.6 by adding Isopropyl β-D-1-thiogalactopyranoside (IPTG) to a final concentration of 1 mM. Cells were harvested 3 h after induction by centrifugation. Cell pellets were resuspended in the lysis buffer (Tris-HCl pH 8.0 50 mM, NaCl 500 mM, DTT 1 mM, PMSF 1 mM, EDTA 1 mM, glycerol 5% (v/v)) supplemented with an EDTA-free antiprotease tablet (Roche) and lysed by sonication. Cell lysates were centrifuged for 30 min at 35,000×*g*. All chromatography purifications were performed on an ÄKTA Pure purification system (Cytiva) at 4 °C. The cell lysate supernatant was loaded on a Ni-NTA column, and the protein of interest was eluted in an imidazole gradient in buffers containing 1 M of NaCl. Fractions containing the protein TruB and TrmB were pooled and dialyzed against the storage buffer (Tris-HCl pH 8.0 50 mM, NaCl 150 mM, DTT 2 mM, EDTA 0.5 mM). TrmA fractions were pooled and dialyzed against a high-salt storage buffer (Tris-HCl pH 8.0 50 mM, NaCl 750 mM, DTT 2 mM, EDTA 0.5 mM) to protect the N-terminal end from cleavage. The N-terminal His-tag was retained because the recombinant proteins showed neither aggregation nor solubility issues. Dialyzed protein samples of TrmB, TruB and TrmA were then concentrated with an Amicon 50,000 MWCO (Millipore) to approximately 10 mg/ml, 25 mg/ml and 30 mg/ml, respectively, and stored at −20 °C. Their respective protein concentrations were determined by absorbance at 280 nm using mM extinction coefficients of 28.0 mM^−1^ cm^−1^, 20.9 mM^−1^ cm^−1^ and 35.7 mM^−1^ cm^−1^, respectively.

### RNA sample preparation for NMR and kinetic assays

The *in vitro* transcription and purification of unmodified *E. coli* tRNA^Asp(GUC)^, tRNA^Val(UAC)^, and tRNA^Phe(GAA)^ used for NMR experiments and enzymatic activity assays, as well as the production of the modified tRNA samples used for NMR assignment and structural comparison, have been described in detail in a report describing the chemical shift assignment of these tRNAs (32).

### NMR spectroscopy

2D (^1^H,^15^N)-BEST-TROSY spectra of unmodified and modified *E. coli* tRNA^Asp^ and tRNA^Val^ were measured at 311 K and 298 K, respectively, on a Bruker AVIII-HD 700 MHz spectrometer; and 2D (^1^H,^15^N)-BEST-TROSY spectra of unmodified and modified *E. coli* tRNA^Phe^ were measured at 311 K on a Bruker AVIII-HD 950 MHz spectrometer, all with 5-mm Shigemi tubes and in the NMR buffer (10 mM Na-phosphate pH 6.5, 10 mM MgCl_2_) supplemented with 5% (v/v) D_2_O. These spectra were used for the assignment of the imino resonance, for comparing the structural properties of unmodified and modified sample and for identifying the NMR signature of individual modifications. For monitoring the maturation of tRNA^Phe^, tRNA^Val^, and tRNA^Asp^ in *E. coli* extracts, NMR spectra were measured on a Bruker AVIII-HD 700 MHz spectrometer at 30 °C with each of the unmodified ^15^N-[U/G]-labeled tRNA^Phe^, tRNA^Val^, and tRNA^Asp^ at 80 μM in *E. coli* extracts supplemented with NaH_2_PO_4_/K_2_HPO_4_ pH 6.5 150 mM, NH_4_Cl 5 mM, MgCl_2_ 5 mM, DTT 2 mM, EDTA 0.1 mM, SAM 4 mM, ATP 4 mM, NADPH 4 mM, and D_2_O 5% (v/v). Concentrations of tRNAs were determined based on their respective extinction coefficient at 260 nm, *i.e.* 828 mM^−1^ cm^−1^, 823 mM^−1^ cm^−1^, and 819 mM^−1^ cm^−1^, for tRNA^Phe^, tRNA^Val^, and tRNA^Asp^, respectively. We selected an 80 μM concentration of tRNA as a compromise that provides a sufficient signal-to-noise ratio in NMR measurements while remaining close to cellular tRNA concentrations. Each 2D (^1^H,^15^N)-BEST-TROSY experiment of the series was measured with a recycling delay of 200 ms, a SW (^15^N) of 26 ppm, and 64 increments of 148 scans for a total experimental time of 60 min. The data were processed using TOPSPIN 3.6 (Bruker) and analysed with NMRFAM-SPARKY (48). For the time-dependent quantitative analysis of NMR peaks, peak height values were extracted from the NMR maturation experiments using NMRFAM-SPARKY. These values were normalized to a selected high-intensity, stable reference peak for each tRNA. In the tRNA^Phe^, tRNA^Val^, and tRNA^Asp^ experiments, normalization was performed using signals of G71, U64, and U11, respectively. Uncertainties were estimated from spectral noise, with the RMS of the noise averaged across multiple regions and spectra within the same experimental series (the same time-resolved experiment, namely, the maturation of a given tRNA in a given extract). The resulting mean RMS noise was applied to the entire experimental series. Reported error bars represent the uncertainty in peak heights, calculated as ±2 × the normalized mean RMS of the noise, thereby approximating a 95% confidence interval (CI 95%).

### TrmA and TrmB enzymatic assays

To measure T54 and m^7^G46 formation by TrmA and TrmB, respectively, 10 μM of unmodified tRNA^Phe^, unmodified tRNA^Val^ and unmodified tRNA^Asp^, were incubated each in a 50 μl reaction with 300 nM of enzyme, 20 μM non-radioactive SAM and 50 nM of radioactive [^3^H]-SAM. Reactions were performed in the maturation buffer (NaH_2_PO_4_/K_2_HPO_4_ pH 7.0 100 mM, NH_4_Cl 5 mM, MgCl_2_ 1.5 mM, DTT 2 mM, EDTA 0.1 mM) and were incubated at 37 °C. Samples were quenched at t = 10 min by adding 5% (v/v) cold trichloracetic acid (TCA). Quenched samples were filtered through Whatman glass microfibers disks pre-soaked with 5% (v/v) TCA, washed four times with 5% (v/v) TCA, and one final time with ethanol. The filter disks were dried, then 5 ml Optiphase 'HISAFE' 2 scintillation cocktail (PerkinElmer) were added, and the counts per minute (CPM) equivalent to the incorporated [^3^H]-methyl were determined by scintillation counting. Then CPM values were converted to concentrations of modified tRNAs using [^3^H]-SAM/CPM calibration standards (Fig. S6*C*). Enzymatic reactions were performed in four independent replicates (N = 4). Standard deviations were relatively uniform across tRNA substrates and correspond to 8 to 14% for TrmA and 16 to 22% for TrmB. To ensure that the reactions were still in the initial linear phase of product formation, time-course experiments were also performed under the same experimental conditions. For TrmB (15-min incubation), 50 μl aliquots were taken at five selected time points, with 3-min intervals (Fig. S6*A*). For TrmA (10-min incubation), aliquots were taken at five selected time points, with 2-min intervals (Fig. S6*A*). Samples were treated as described above.

## Data availability

Data are provided in the BMRB public repository, in the manuscript and its associated supplementary information.

## Supporting information

This article contains supporting information (47).

## Conflict of interest

The authors declare that they do not have any conflicts of interest with the content of this article.

## References

[1] Katz A., Elgamal S., Rajkovic A., Ibba M. (2016). Non-canonical roles of tRNAs and tRNA mimics in bacterial cell biology. Mol. Microbiol..

[2] Su Z., Wilson B., Kumar P., Dutta A. (2020). Noncanonical roles of tRNAs: tRNA fragments and beyond. Annu. Rev. Genet..

[3] Phizicky E.M., Hopper A.K. (2023). The life and times of a tRNA. RNA.

[4] Berg M.D., Brandl C.J. (2021). Transfer RNAs: diversity in form and function. RNA Biol..

[5] Barraud P., Tisné C. (2019). To be or not to be modified: miscellaneous aspects influencing nucleotide modifications in tRNAs. IUBMB Life.

[6] Hopper A.K. (2013). Transfer RNA post-transcriptional processing, turnover, and subcellular dynamics in the yeast Saccharomyces cerevisiae. Genetics.

[7] Cappannini A., Ray A., Purta E., Mukherjee S., Boccaletto P., Moafinejad S.N. (2024). MODOMICS: a database of RNA modifications and related information. 2023 update. Nucleic Acids Res..

[8] Jackman J.E., Alfonzo J.D. (2013). Transfer RNA modifications: nature's combinatorial chemistry playground. Wiley Interdiscip. Rev. RNA.

[9] de Crécy-Lagard V., Jaroch M. (2021). Functions of bacterial tRNA modifications: from ubiquity to diversity. Trends Microbiol..

[10] Motorin Y., Helm M. (2010). tRNA stabilization by modified nucleotides. Biochemistry.

[11] Lorenz C., Lünse C.E., Mörl M. (2017). tRNA modifications: impact on structure and thermal adaptation. Biomolecules.

[12] Alexandrov A., Chernyakov I., Gu W., Hiley S.L., Hughes T.R., Grayhack E.J. (2006). Rapid tRNA decay can result from lack of nonessential modifications. Mol. Cell.

[13] Kimura S., Waldor M.K. (2019). The RNA degradosome promotes tRNA quality control through clearance of hypomodified tRNA. Proc. Natl. Acad. Sci. U. S. A..

[14] Smith T.J., Giles R.N., Koutmou K.S. (2024). Anticodon stem-loop tRNA modifications influence codon decoding and frame maintenance during translation. Semin. Cell Dev. Biol..

[15] Agris P.F., Narendran A., Sarachan K., Väre V.Y.P., Eruysal E. (2017). The importance of being modified: the role of RNA modifications in translational fidelity. Enzymes.

[16] Yared M.-J., Marcelot A., Barraud P. (2024). Beyond the anticodon: tRNA core modifications and their impact on structure, translation and stress adaptation. Genes (Basel).

[17] Grosjean H., Droogmans L., Roovers M., Keith G. (2007). Detection of enzymatic activity of transfer RNA modification enzymes using radiolabeled tRNA substrates. Methods Enzymol..

[18] Han L., Phizicky E.M. (2018). A rationale for tRNA modification circuits in the anticodon loop. RNA.

[19] Sokołowski M., Klassen R., Bruch A., Schaffrath R., Glatt S. (2018). Cooperativity between different tRNA modifications and their modification pathways. Biochim. Biophys. Acta Gene Regul. Mech..

[20] Tomikawa C., Yokogawa T., Kanai T., Hori H. (2010). N7-Methylguanine at position 46 (m7G46) in tRNA from Thermus thermophilus is required for cell viability at high temperatures through a tRNA modification network. Nucleic Acids Res..

[21] Ishida K., Kunibayashi T., Tomikawa C., Ochi A., Kanai T., Hirata A. (2011). Pseudouridine at position 55 in tRNA controls the contents of other modified nucleotides for low-temperature adaptation in the extreme-thermophilic eubacterium Thermus thermophilus. Nucleic Acids Res..

[22] Gato A., Catala M., Tisné C., Barraud P. (2021). A method to monitor the introduction of posttranscriptional modifications in tRNAs with NMR spectroscopy. Methods Mol. Biol..

[23] Barraud P., Gato A., Heiss M., Catala M., Kellner S., Tisné C. (2019). Time-resolved NMR monitoring of tRNA maturation. Nat. Commun..

[24] Yared M.-J., Yoluç Y., Catala M., Tisné C., Kaiser S., Barraud P. (2023). Different modification pathways for m1A58 incorporation in yeast elongator and initiator tRNAs. Nucleic Acids Res..

[25] Meyer B., Immer C., Kaiser S., Sharma S., Yang J., Watzinger P. (2020). Identification of the 3-amino-3-carboxypropyl (acp) transferase enzyme responsible for acp3U formation at position 47 in Escherichia coli tRNAs. Nucleic Acids Res..

[26] Jones J.D., Franco M.K., Giles R.N., Eyler D.E., Tardu M., Smith T.J. (2024). Conserved 5-methyluridine tRNA modification modulates ribosome translocation. Proc. Natl. Acad. Sci. U. S. A..

[27] Schultz S.K., Katanski C.D., Halucha M., Peña N., Fahlman R.P., Pan T. (2024). Modifications in the T arm of tRNA globally determine tRNA maturation, function, and cellular fitness. Proc. Natl. Acad. Sci. U. S. A..

[28] Schultz S.K.-L., Kothe U. (2020). tRNA elbow modifications affect the tRNA pseudouridine synthase TruB and the methyltransferase TrmA. RNA.

[29] Lucas M.C., Pryszcz L.P., Medina R., Milenkovic I., Camacho N., Marchand V. (2024). Quantitative analysis of tRNA abundance and modifications by nanopore RNA sequencing. Nat. Biotechnol..

[30] Machnicka M.A., Olchowik A., Grosjean H., Bujnicki J.M. (2014). Distribution and frequencies of post-transcriptional modifications in tRNAs. RNA Biol..

[31] Bacusmo J.M., Babor J., Hu J., Cao B., Kellner S., Szkrybalo S. (2024). Synergistic effects of tRNA modification defects in Escherichia coli K12. bioRxiv.

[32] Yared M.-J., Chagneau C., Barraud P. (2024). Imino chemical shift assignments of tRNAAsp, tRNAVal and tRNAPhe from Escherichia coli. Biomol. NMR Assign..

[33] Schultz S.K., Meadows K., Kothe U. (2023). Molecular mechanism of tRNA binding by the Escherichia coli N7 guanosine methyltransferase TrmB. J. Biol. Chem..

[34] de Jesus V., Biedenbänder T., Vögele J., Wöhnert J., Fürtig B. (2022). NMR assignment of non-modified tRNAIle from Escherichia coli. Biomol. NMR Assign..

[35] Catala M., Gato A., Tisné C., Barraud P. (2020). 1H, 15N chemical shift assignments of the imino groups of yeast tRNAPhe: influence of the post-transcriptional modifications. Biomol. NMR Assign..

[36] Puglisi E.V., Puglisi J.D. (2007). Probing the conformation of human tRNA(3)(Lys) in solution by NMR. FEBS Lett..

[37] Biedenbänder T., de Jesus V., Schmidt-Dengler M., Helm M., Corzilius B., Fürtig B. (2022). RNA modifications stabilize the tertiary structure of tRNAfMet by locally increasing conformational dynamics. Nucleic Acids Res..

[38] Wang J., Koduru T., Harish B., McCallum S.A., Larsen K.P., Patel K.S. (2023). Pressure pushes tRNALys3 into excited conformational states. Proc. Natl. Acad. Sci. U. S. A..

[39] Zhang X., Walker R.C., Phizicky E.M., Mathews D.H. (2014). Influence of sequence and covalent modifications on yeast tRNA dynamics. J. Chem. Theor. Comput..

[40] Phizicky E.M., Alfonzo J.D. (2010). Do all modifications benefit all tRNAs?. FEBS Lett..

[41] Chernyakov I., Whipple J.M., Kotelawala L., Grayhack E.J., Phizicky E.M. (2008). Degradation of several hypomodified mature tRNA species in Saccharomyces cerevisiae is mediated by Met22 and the 5'-3' exonucleases Rat1 and Xrn1. Genes Dev..

[42] Dewe J.M., Whipple J.M., Chernyakov I., Jaramillo L.N., Phizicky E.M. (2012). The yeast rapid tRNA decay pathway competes with elongation factor 1A for substrate tRNAs and acts on tRNAs lacking one or more of several modifications. RNA.

[43] De Zoysa T., Phizicky E.M. (2020). Hypomodified tRNA in evolutionarily distant yeasts can trigger rapid tRNA decay to activate the general amino acid control response, but with different consequences. Plos Genet..

[44] Kadaba S., Krueger A., Trice T., Krecic A.M., Hinnebusch A.G., Anderson J. (2004). Nuclear surveillance and degradation of hypomodified initiator tRNAMet in S. cerevisiae. Genes Dev..

[45] Kadaba S., Wang X., Anderson J.T. (2006). Nuclear RNA surveillance in Saccharomyces cerevisiae: Trf4p-dependent polyadenylation of nascent hypomethylated tRNA and an aberrant form of 5S rRNA. RNA.

[46] Tasak M., Phizicky E.M. (2022). Initiator tRNA lacking 1-methyladenosine is targeted by the rapid tRNA decay pathway in evolutionarily distant yeast species. Plos Genet..

[47] Baba T., Ara T., Hasegawa M., Takai Y., Okumura Y., Baba M. (2006). Construction of Escherichia coli K-12 in-frame, single-gene knockout mutants: the Keio collection. Mol. Syst. Biol..

[48] Lee W., Tonelli M., Markley J.L. (2015). NMRFAM-SPARKY: enhanced software for biomolecular NMR spectroscopy. Bioinformatics.

[49] Leontis N.B., Westhof E. (2001). Geometric nomenclature and classification of RNA base pairs. RNA.

