# Supplementary material for: Mapping the temporality and structural impacts of modifications in Escherichia coli tRNAs
Source: J Biol Chem. 2026 Mar 3;302(4):111337. doi: 10.1016/j.jbc.2026.111337 (PMC13052159; doi:10.1016/j.jbc.2026.111337)
Supplement: Supplementary Tables and Figures [file mmc1.pdf]

SUPPORTING INFORMATION

Mapping the temporality and structural impacts of modifications in *E. coli* tRNAs

Marcel-Joseph Yared, Carine Chagneau & Pierre Barraud

Content:

Supplementary Tables S1-S3 pages 2-4
Supplementary Figures S1-S26 pages 5-31

SUPPLEMENTARY TABLES

Supplementary Table S1 – *E. coli* strains used in this study

Strain	Genotype	Source
BW25113 (Keio Parent)	*E. coli* K-12 F-, lacI+, Δ(araD-araB)567, ΔlacZ4787(::rrnB-3), λ-, rph-1, Δ(rhaD-rhaB)568, hsdR514	[1]
BW25113-*trmBΔ* (JW2927)	*E. coli* K-12 F-, lacI+, Δ(araD-araB)567, ΔlacZ4787(::rrnB-3), λ-, ΔyggH735::kan, rph-1, Δ(rhaD-rhaB)568, hsdR514	[1]
BW25113-*dusAΔ* (JW5950)	*E. coli* K-12 F-, lacI+, Δ(araD-araB)567, ΔlacZ4787(::rrnB-3), λ-, rph-1, Δ(rhaD-rhaB)568, ΔdusA743::kan, hsdR514	[1]
BW25113-*dusCΔ* (JW2128)	*E. coli* K-12 F-, lacI+, Δ(araD-araB)567, ΔlacZ4787(::rrnB-3), λ-, ΔdusC767::kan, rph-1, Δ(rhaD-rhaB)568, hsdR514	[1]
BW25113-*trmAΔ* (JW3937)	*E. coli* K-12 F-, lacI+, Δ(araD-araB)567, ΔlacZ4787(::rrnB-3), λ-, rph-1, Δ(rhaD-rhaB)568, ΔtrmA753::kan, hsdR514	[1]
BW25113-*truBΔ* (JW3135)	*E. coli* K-12 F-, lacI+, Δ(araD-araB)567, ΔlacZ4787(::rrnB-3), λ-, ΔtruB778::kan, rph-1, Δ(rhaD-rhaB)568, hsdR514	[1]
BW25113-*yfiPΔ* (JW5409)	*E. coli* K-12 F-, lacI+, Δ(araD-araB)567, ΔlacZ4787(::rrnB-3), λ-, ΔyfiP751::kan, rph-1, Δ(rhaD-rhaB)568, hsdR514	[1]
BW25113-*thiIΔ* (JW0413)	*E. coli* K-12 F-, lacI+, Δ(araD-araB)567, ΔlacZ4787(::rrnB-3), ΔthiI780::kan, λ-, rph-1, Δ(rhaD-rhaB)568, hsdR514	[1]
BW25113-*dusBΔ* (JW3228)	*E. coli* K-12 F-, lacI+, Δ(araD-araB)567, ΔlacZ4787(::rrnB-3), λ-, ΔdusB778::kan, rph-1, Δ(rhaD-rhaB)568, hsdR514	[1]
BW25113-*truCΔ* (JW2762)	*E. coli* K-12 F-, lacI+, Δ(araD-araB)567, ΔlacZ4787(::rrnB-3), λ-, ΔtruC789::kan, rph-1, Δ(rhaD-rhaB)568, hsdR514	[1]
BL21(DE3)	*E. coli* B F- ompT hsdSB (rB–, mB–) gal dcm (DE3)	Agilent

[1] Baba, T., Ara, T., Hasegawa, M., Takai, Y., Okumura, Y., Baba, M., Datsenko, K. A., Tomita, M., Wanner, B. L., and Mori, H. (2006) Construction of Escherichia coli K-12 in-frame, single-gene knockout mutants: the Keio collection. *Mol Syst Biol* **2**, 2006.0008.

Supplementary Table S2 – List of primers used in this study

TruB-M1_NheI_Fwd: GCC-ACG-GCT-AGC-AGT-CGT-CCT-CGT-CGT-CGC
TruB-A314_BamHI_Rev: GCC-ACG-GGA-TCC-TTA-CGC-CGG-GTA-TTC-AAC-CAC

TrmB_M1_NheI_Fwd: GCC-AGA-GCT-AGC-AAA-AAC-GAC-GTC-ATT-TCA-CCG-G
TrmB_K239_BamHI_Rev: GCC-ACC-GGA-TCC-TTA-TTT-CAC-CCT-CT-CGA-ACA-TTA-AGT-CCC

TrmA_M1_NheI_Fwd: GGC-ACC-GCT-AGC-ACC-CCC-GAA-CAC-CTT-CCA-AC
TrmA_K366_BamHI_Rev: GCC-ACG-GGA-TCC-TTA-CTT-CGC-GGT-CAG-TAA-TAC-GC

truB_A: GAC-GTT-CCT-CAA-CGA-CAA-AGA
truB_B: TGT-CGG-AGT-CCA-GCA-GAT-AC
trmA_A: GGG-CTT-GGA-GGG-GTA-TCT-AA
trmA_B: ATA-CAG-GTC-ATC-GCC-ATC-GT
yfiP_A: AGA-GCG-TAG-CGG-TAA-AGT-GC
yfiP_B: GCA-ACG-GTA-TCA-GGC-AAA-AT
trmB_A: GCC-ATA-CTT-TGT-AGG-GCG-TCT
trmB_B: AAC-CAA-TCT-CAA-GCG-TCA-CC
dusA_A: TCG-TCG-AAC-CTG-GAT-TGT-TT
dusA_B: CAA-CGC-TAC-CGG-ATG-TTC-TT
dusC_A: CAC-CCC-AGA-TGA-GAT-GGA-TT
dusC_B: CCA-TTG-TGG-GAA-CTG-ACC-TAA
thiI_A: CGC-AAT-GGT-AAG-GTG-ATG-TG
thiI_B: CAG-AAG-GTT-TTG-CCT-TCC-AG
dusB_A: CGC-CGC-CTT-GCA-GTC-ACA-GT
dusB_B: GCG-AAT-ACC-GGG-TTC-ATC
truC_A: GAT-CCC-GCG-AAT-GCA-CGA-T
truC_B: GCC-AGC-AGC-CGT-CCG-GCC-TC
k1: CAG-TCA-TAG-CCG-AAT-AGC-CT
k2: CGG-TGC-CCT-GAA-TGA-ACT-GC

T7 promoter: 5’TAA-TAC-GAC-TCA-CTA-TAG 3’

E. coli $tRNA^{Phe}$ DNA template :
5’TGGTGCCCGGACTCGGAATCGAACCAAGGACACGGGGATTTTCAATCCCCTGCTCTACCGACTGAGCTATCCGGGCTATAGTGAGTCGTATTA 3’

E. coli $tRNA^{Val}$ DNA template :
5’TGGTGGGTGATGACGGGATCGAACCGCCGACCCCCTCCTTGTAAGGGAGGTGCTCTCCCAGCTGAGCTAATCACCCTATAGTGAGTCGTATTA 3’

E. coli $tRNA^{Asp}$ DNA template :
5’TGGCGGAACGGACGGGACTCGAACCCGCGACCCCCTGCGTGACAGGCAGGTATTCTAACCGACTGAACTACCGCTCCTATAGTGAGTCGTATTA 3’

Supplementary Table S3 – *E. coli* enzymes responsible of the modification of the tRNAs used in this study

**Modification**	**Enzyme**	**Uniprot entry**
Ψ39	TruA	P07649
Ψ55	TruB	P60340
Ψ65	TruC	P0AA41
T54	TrmA	P23003
m^7G46	TrmB	P0A8I5
s^4U8	ThiI	P77718
acp^3U47	YfiP	Q47319
D20/D20a	DusA	P32695
D17	DusB	P0ABT5
D16	DusC	P33371

SUPPLEMENTARY FIGURES

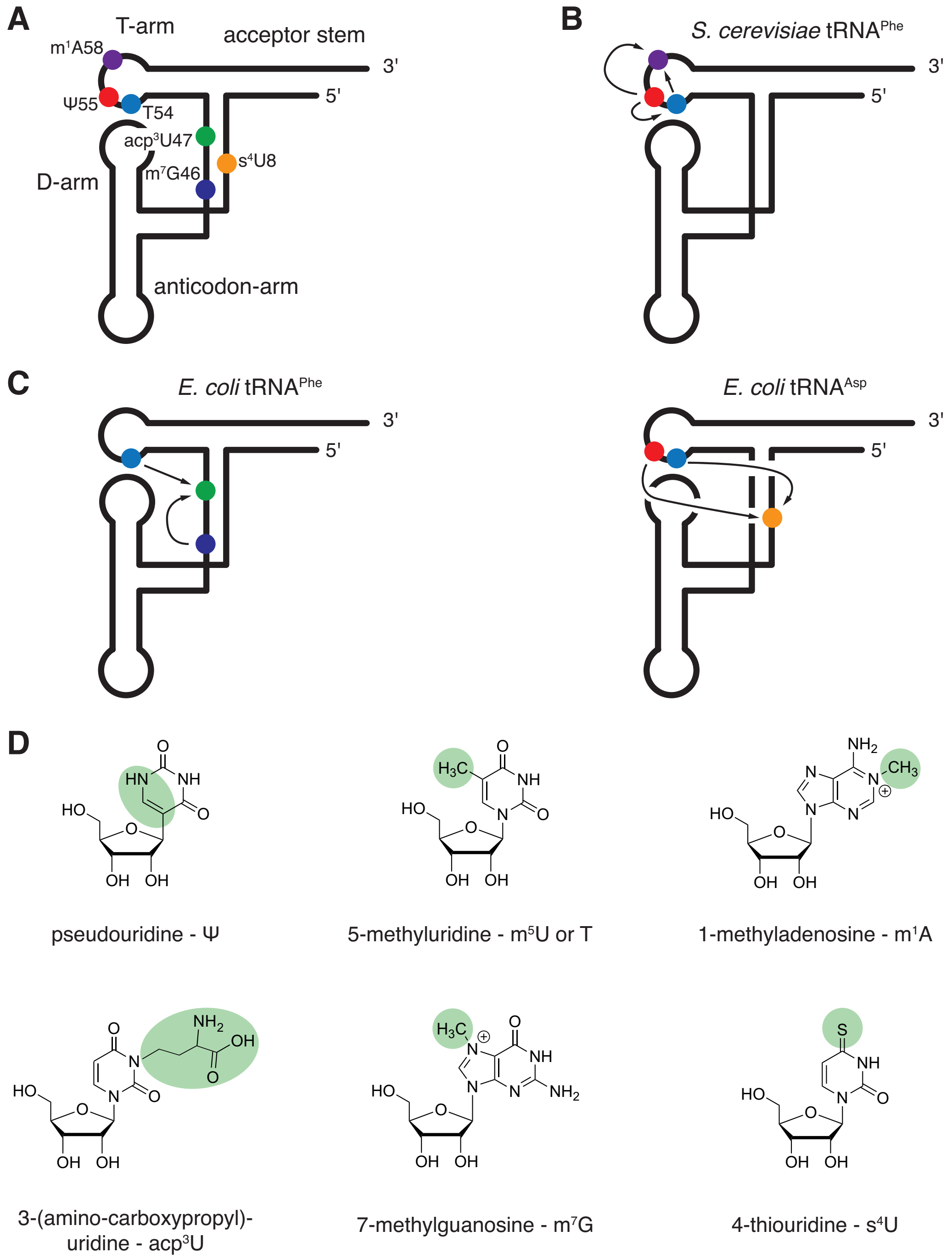

Supplementary Figure S1: Modifications in the tRNA core and modification circuits

(**A**) Schematic representation of the L-shaped tertiary structure of tRNA. The acceptor stem stacks on the T-arm, and the D-arm stacks on the anticodon-arm. Common modified nucleotides of the tRNA core region are displayed with a specific color. (**B**) Modification circuits in the T-arm of yeast $tRNA^{Phe}$. (**C**) Modification circuits in *E. coli* $tRNA^{Phe}$ and *E. coli* $tRNA^{Asp}$. The same color code is used as in (A). (**D**) Chemical structure of common modified nucleotides of the tRNA core region: pseudouridine (Ψ), 5-methyluridine (m^5U or T), 1-methyladenosine (m^1A), 3-(amino-carboxypropyl)-uridine (acp^3U), 7-methylguanosine (m^7G), and 4-thiouridine (s^4U). Modifications are highlighted in green.

A

```
        1          10            20            30            40            50            60            70
tRNAPhe GCCCGGAUAGCUCAGUCGGU-AGAGCAGGGGAUUGAAAAUCCCCGUGUCCUUGGUUCGAUUCCGAGUCCGGGCACCA
tRNAVal GGGUGAUUAGCUCAGCUGGG-AGAGCACCUCCCUUACAAGGAGGGGGUCGGCGGUUCGAUCCCGUCAUCACCCACCA
tRNAAsp GGAGCGGUAGUUCAGUCGGUUAGAAUACCUGCCUGUCACGCAGGGGGUCGCGGGUUCGAGUCCCGUCCGUUCCGCCA
```

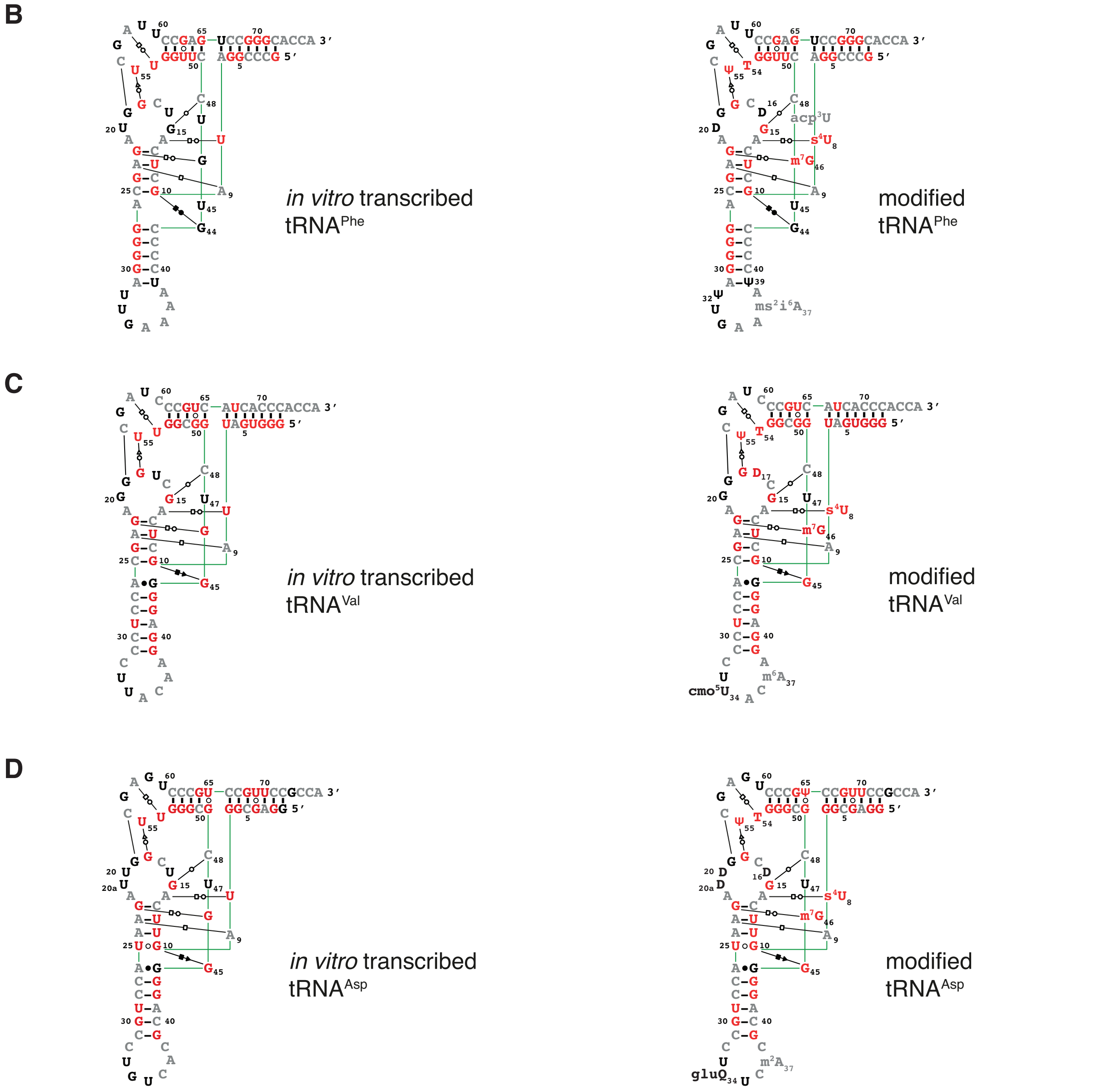

Supplementary Figure S2: Summary of sample chemical shift assignment

(**A**) Sequence alignment with nucleotide numbering of unmodified *E. coli* tRNA^{Phe}, tRNA^{Val}, and tRNA^{Asp}. (**B**) Summary of imino chemical shift assignment of *in vitro* transcribed (left) and modified (right) *E. coli* tRNA^{Phe}. Assigned G and U are in red, unassigned G and U are in black, and A and C, which do not carry imino groups, are in grey. (**C**) Summary of imino chemical shift assignment of *in vitro* transcribed (left) and modified (right) *E. coli* tRNA^{Val}. (**D**) Summary of imino chemical shift assignment of *in vitro* transcribed (left) and modified (right) *E. coli* tRNA^{Asp}. Same colors are used as in (B).

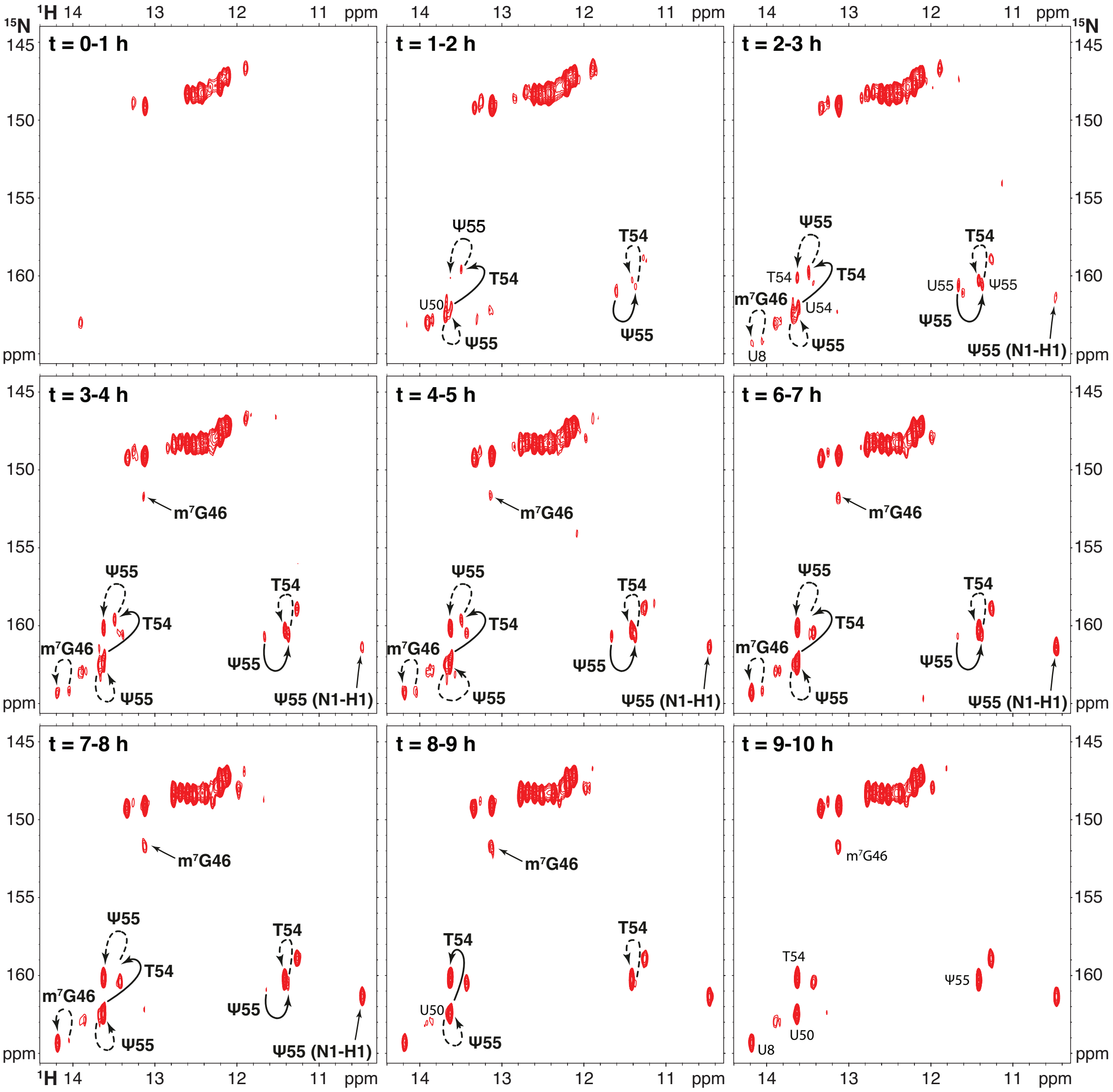

Supplementary Figure S3: Time-resolved NMR monitoring of tRNA^{Phe} maturation.

Imino (^{1}H,^{15}N) correlation spectra of a ^{15}N-labeled tRNA^{Phe} measured in a time-resolved fashion during a continuous incubation in wild-type *E. coli* extract at 30°C. Each NMR spectrum measurement spreads over a 1 h time period, as indicated. Detected modifications are reported with continuous line arrows for direct effects, or dashed arrows for indirect effects. See also Figure 3.

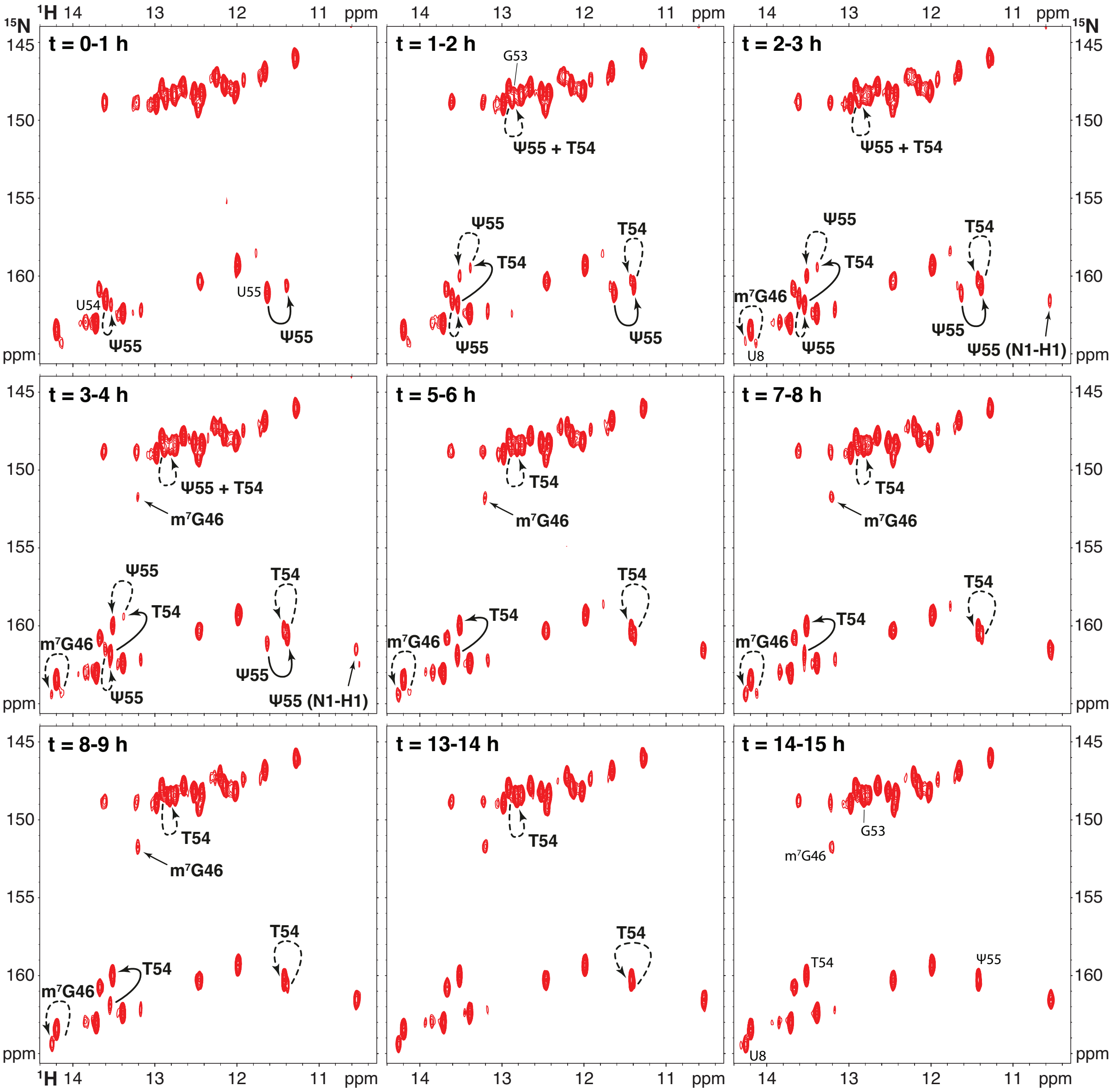

Supplementary Figure S4: Time-resolved NMR monitoring of tRNA^{Val} maturation.

Imino (^{1}H,^{15}N) correlation spectra of a ^{15}N-labeled tRNA^{Val} measured in a time-resolved fashion during a continuous incubation in wild-type *E. coli* extract at 30°C. Each NMR spectrum measurement spreads over a 1 h time period, as indicated. Detected modifications are reported with continuous line arrows for direct effects, or dashed arrows for indirect effects. See also Figure 4.

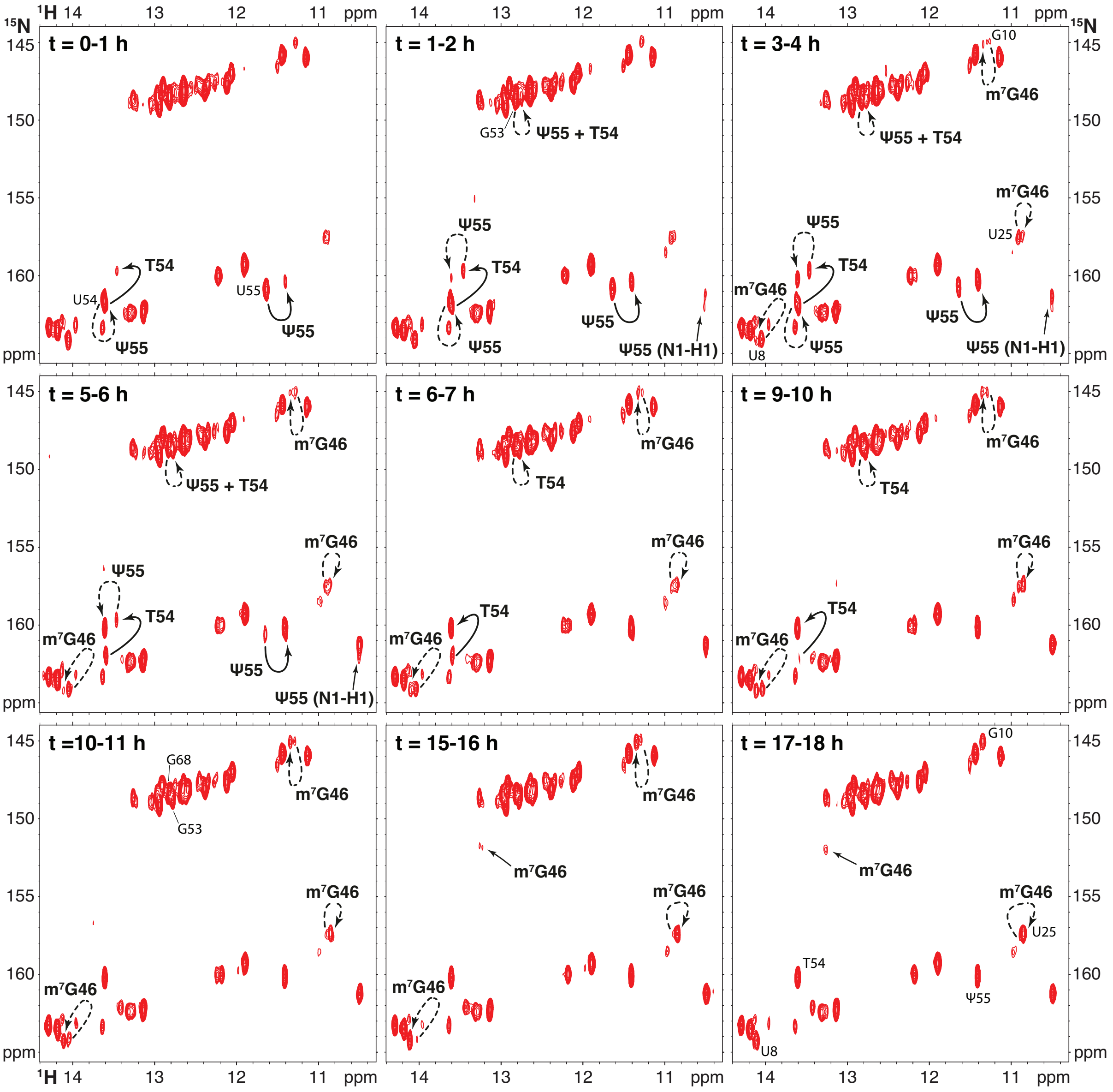

Supplementary Figure S5: Time-resolved NMR monitoring of tRNA^{Asp} maturation.

Imino (^{1}H,^{15}N) correlation spectra of a ^{15}N-labeled tRNA^{Asp} measured in a time-resolved fashion during a continuous incubation in wild-type *E. coli* extract at 30°C. Each NMR spectrum measurement spreads over a 1 h time period, as indicated. Detected modifications are reported with continuous line arrows for direct effects, or dashed arrows for indirect effects. See also Figure 5.

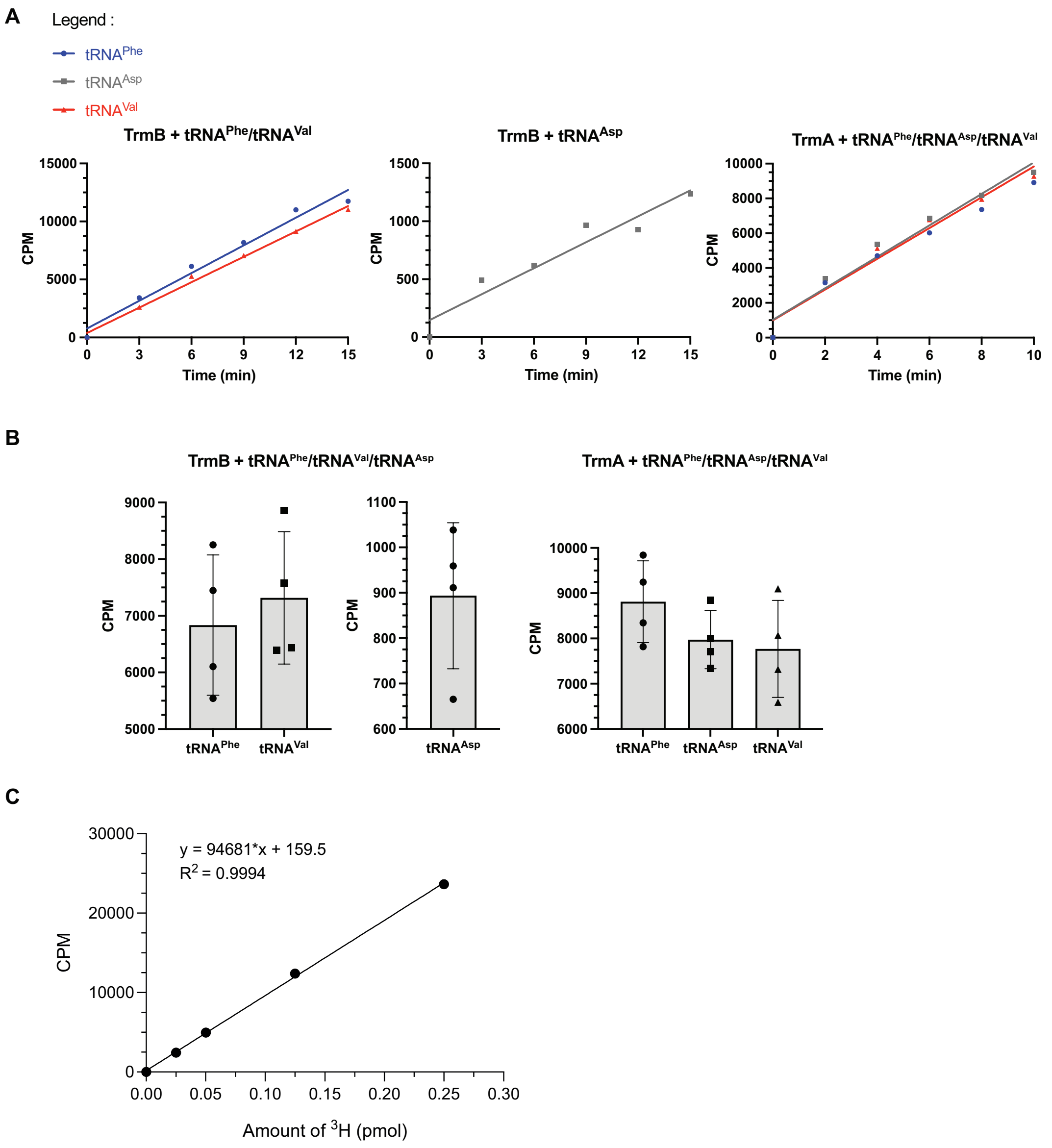

Supplementary Figure S6: Raw kinetic data of TrmB and TrmA reactions with different tRNA substrates

(**A**) Raw data (counts per minute – CPM) corresponding to time-course measurements for m^7G46 and T54 introduction by TrmB and TrmA, respectively. Activity assays were performed on unmodified *E. coli* $tRNA^{Phe}$ (blue circle), $tRNA^{Val}$ (red triangle) and $tRNA^{Asp}$ (grey squares) in independent reactions. (**B**) Raw CPM data corresponding to m^7G46 and T54 formation at t = 10 minutes of incubation by TrmB and TrmA, respectively. Modification assays were performed on *E. coli* $tRNA^{Phe}$, $tRNA^{Val}$ and $tRNA^{Asp}$ in independent reactions. Individual data points are shown, and error bars represent the standard deviation (SD) of four independent replicates (N=4). (**C**) Calibration curve showing the linear relationship between the detected signal in counts per minute (CPM) and the amount of radioactive tritium (^3H). Linear regression analysis yielded the equation y = 94681*x + 159.5, with a coefficient of determination of R^2 = 0.9994. Considering the proportion of radioactive and non-radioactive SAM (ratio = 400), and reaction volume ($V_R = 50*10^{-6}$ L) (see experimental procedures), the conversion of CPM to concentration of modified tRNAs is obtained with the following formula: [modified tRNAs] (pM) = (((y-159,51)/94681)*400) / V_R (L).

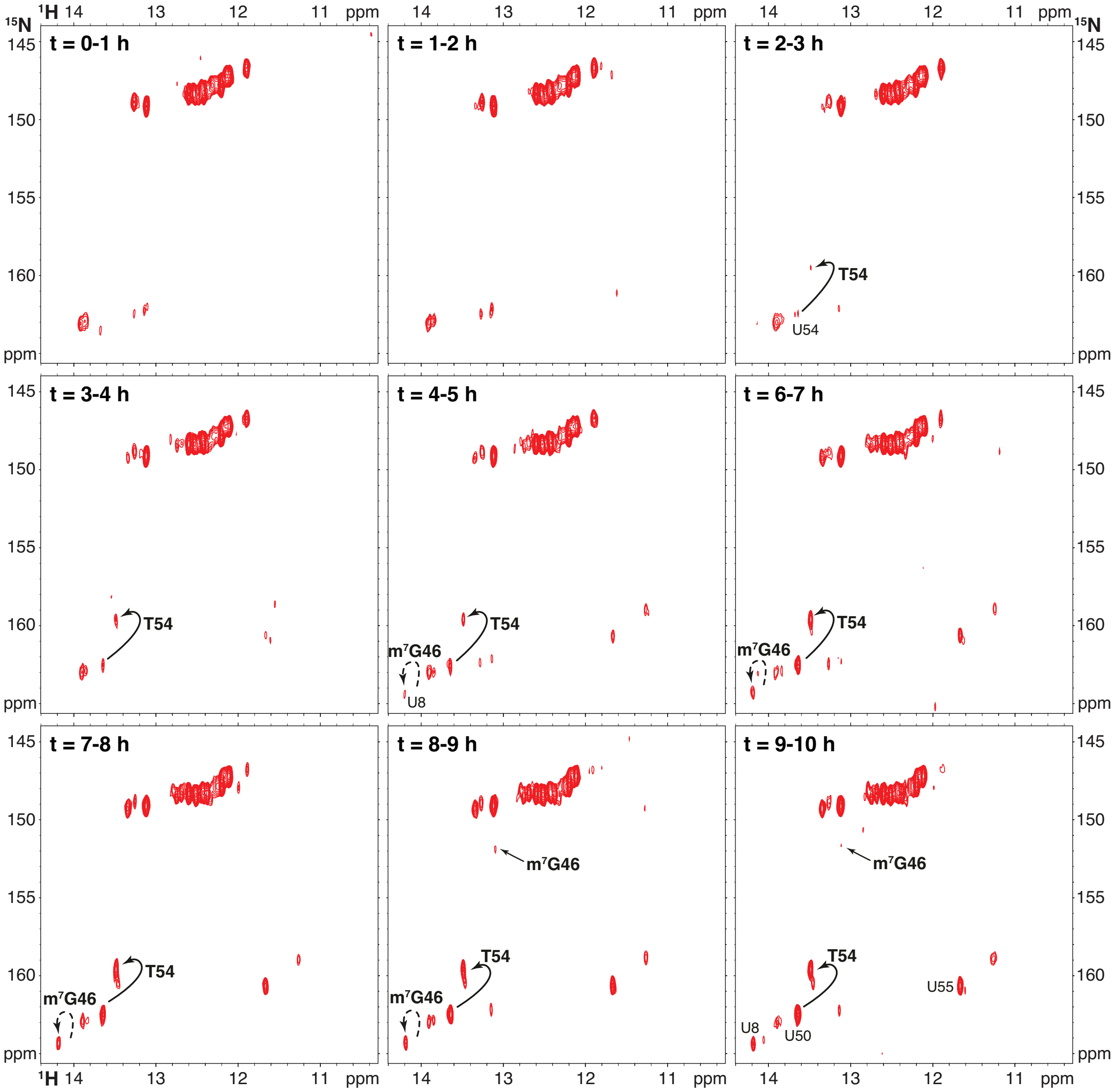

Supplementary Figure S7: Time-resolved NMR monitoring of *E. coli* tRNA^{Phe} maturation in *truBΔ* cell extracts

(*spectra from top to bottom right*) Imino (^{1}H,^{15}N) correlation spectra of a ^{15}N-labelled tRNA^{Phe} measured in a time-resolved fashion during a continuous incubation at 30°C in *E. coli* extract from a *truBΔ* strain over 10 h. TruB is responsible for Ψ55 formation. Each NMR spectrum measurement corresponds to a 1 hour time period, as indicated on the top-left corner of each spectrum. Detected modifications are reported with continuous line arrows for direct effects, or dashed arrows for indirect effects.

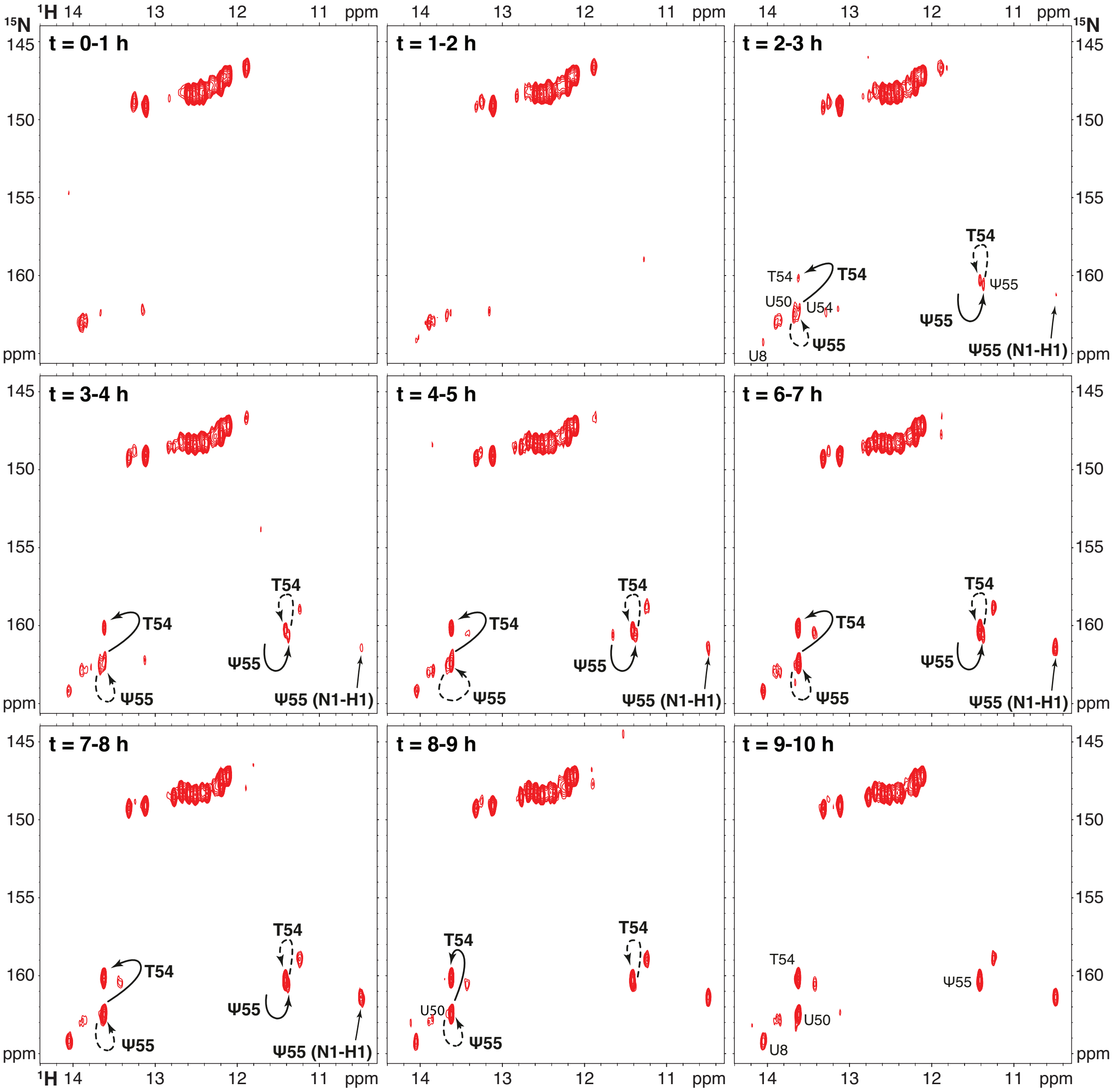

Supplementary Figure S8: Time-resolved NMR monitoring of *E. coli* tRNA^{Phe} maturation in *trmBΔ* cell extracts

(*spectra from top to bottom right*) Imino (^{1}H,^{15}N) correlation spectra of a ^{15}N-labelled tRNA^{Phe} measured in a time-resolved fashion during a continuous incubation at 30°C in *E. coli* extract from a *trmBΔ* strain over 10 h. TrmB is responsible for m^{7}G46 formation. Each NMR spectrum measurement corresponds to a 1 hour time period, as indicated on the top-left corner of each spectrum. Detected modifications are reported with continuous line arrows for direct effects, or dashed arrows for indirect effects.

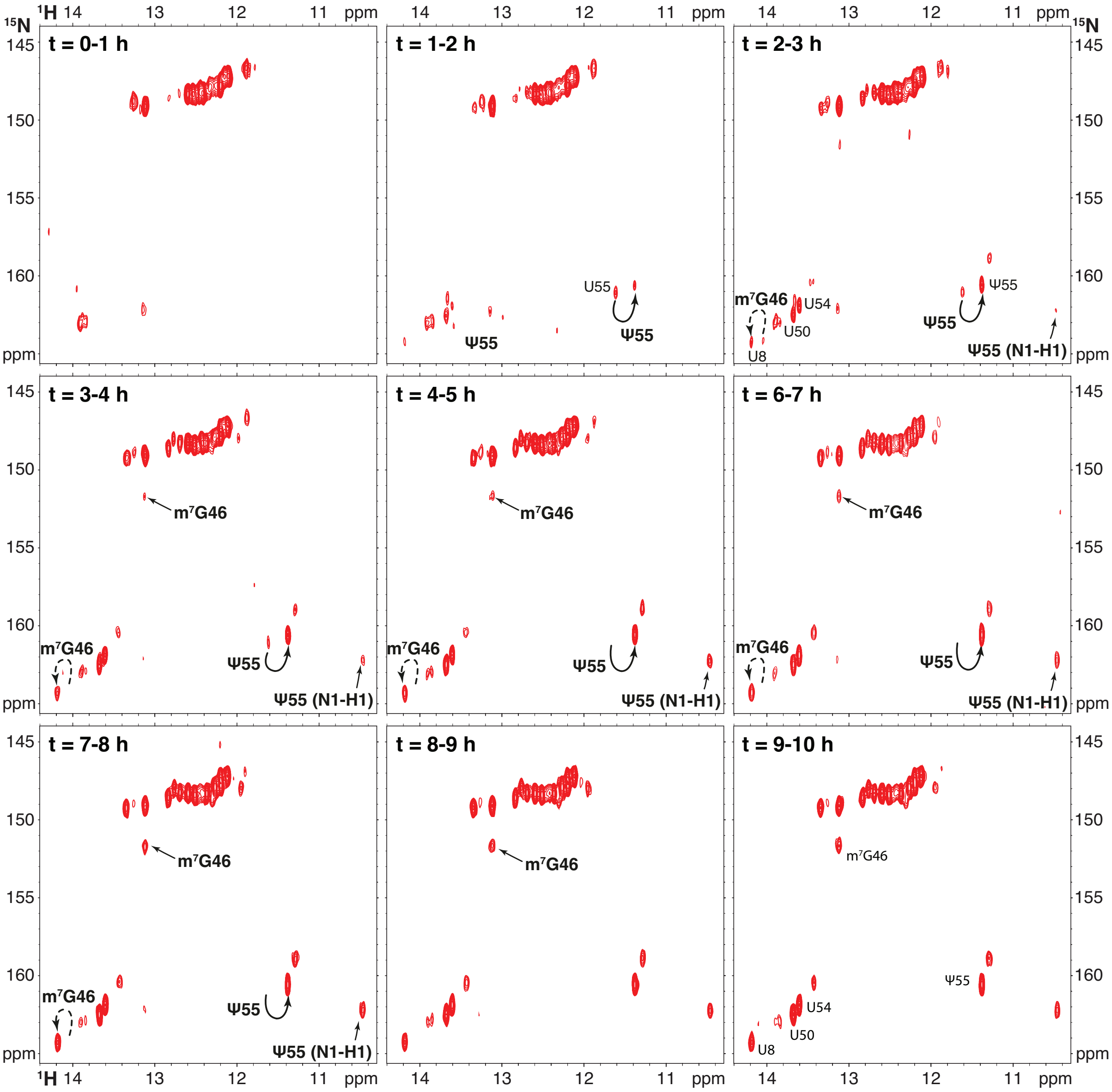

Supplementary Figure S9: Time-resolved NMR monitoring of *E. coli* tRNA^{Phe} maturation in *trmAΔ* cell extracts

(*spectra from top to bottom right*) Imino (^{1}H,^{15}N) correlation spectra of a ^{15}N-labelled tRNA^{Phe} measured in a time-resolved fashion during a continuous incubation at 30°C in *E. coli* extract from a *trmAΔ* strain over 10 h. TrmA is responsible for T54 formation. Each NMR spectrum measurement corresponds to a 1 hour time period, as indicated on the top-left corner of each spectrum. Detected modifications are reported with continuous line arrows for direct effects, or dashed arrows for indirect effects.

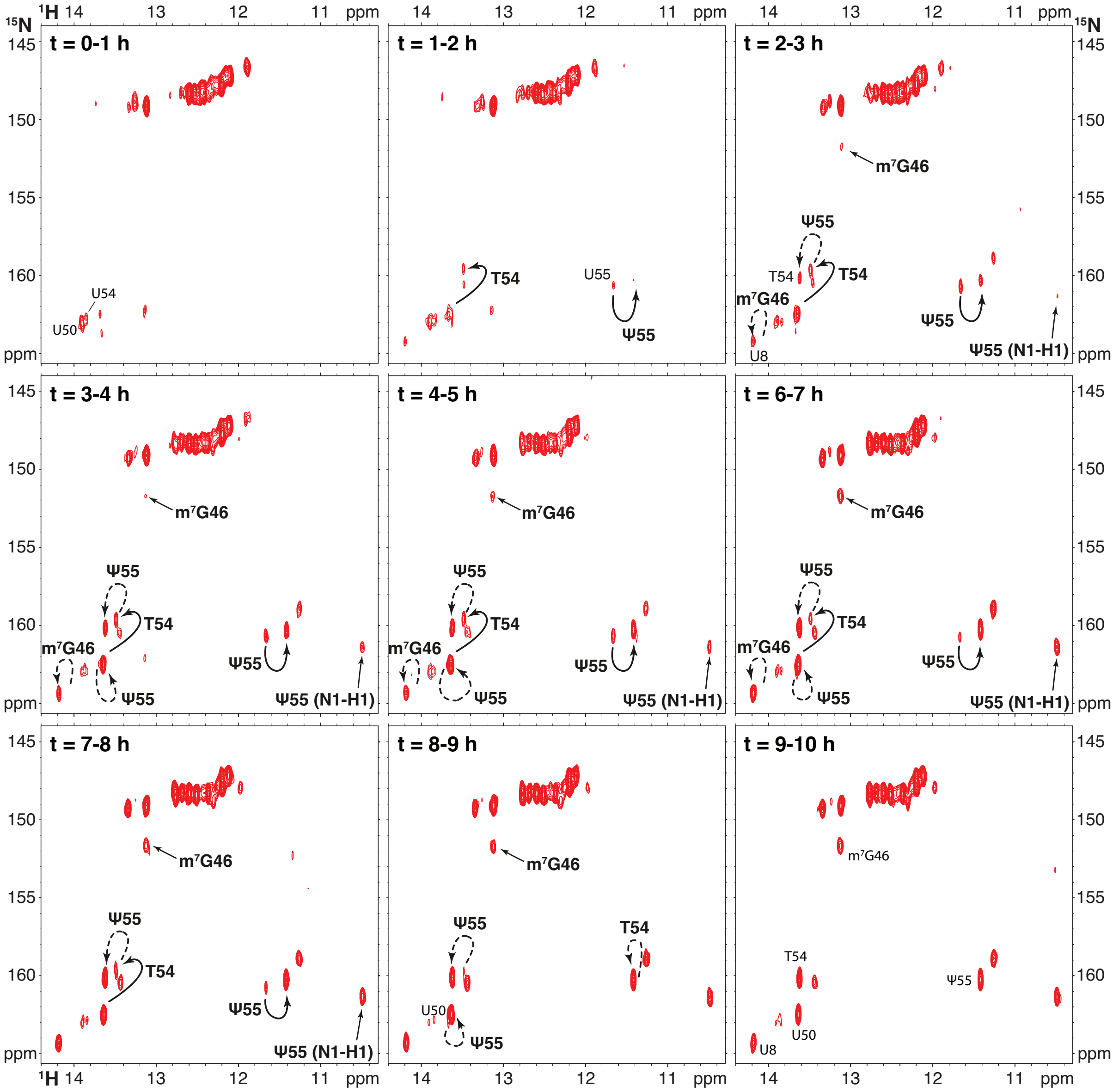

Supplementary Figure S10: Time-resolved NMR monitoring of *E. coli* tRNA^{Phe} maturation in *thiIΔ* cell extracts

(*spectra from top to bottom right*) Imino (^{1}H,^{15}N) correlation spectra of a ^{15}N-labelled tRNA^{Phe} measured in a time-resolved fashion during a continuous incubation at 30°C in *E. coli* extract from a *thiIΔ* strain over 10 h. ThiI is responsible for s^{4}U8 formation. Each NMR spectrum measurement corresponds to a 1 hour time period, as indicated on the top-left corner of each spectrum. Detected modifications are reported with continuous line arrows for direct effects, or dashed arrows for indirect effects.

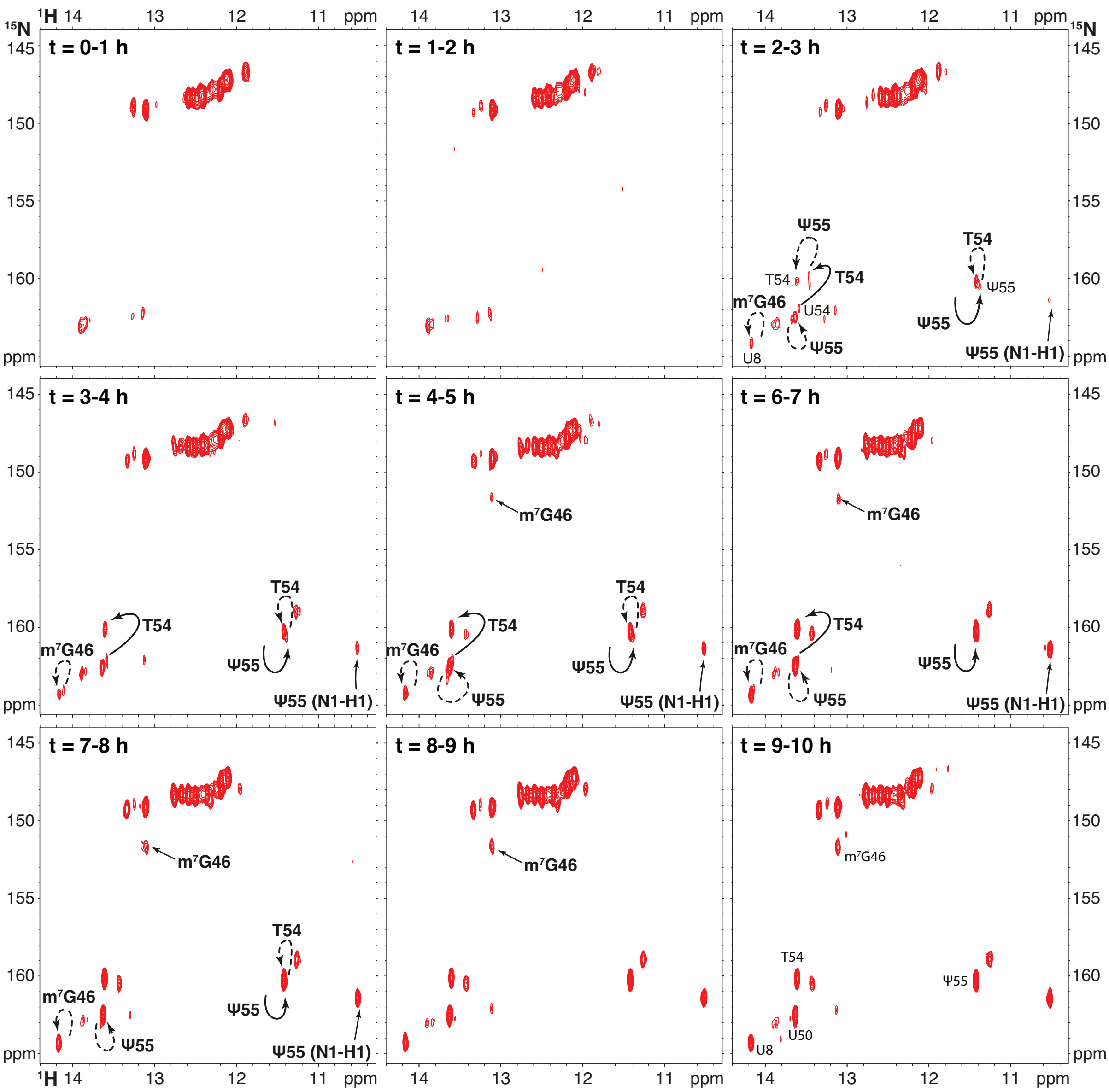

Supplementary Figure S11: Time-resolved NMR monitoring of *E. coli* tRNA^{Phe} maturation in *yfiPΔ* cell extracts

(*spectra from top to bottom right*) Imino (^{1}H,^{15}N) correlation spectra of a ^{15}N-labelled tRNA^{Phe} measured in a time-resolved fashion during a continuous incubation at 30°C in *E. coli* extract from a *yfiPΔ* strain over 10 h. YfiP is responsible for acp^{3}U47 formation. Each NMR spectrum measurement corresponds to a 1 hour time period, as indicated on the top-left corner of each spectrum. Detected modifications are reported with continuous line arrows for direct effects, or dashed arrows for indirect effects.

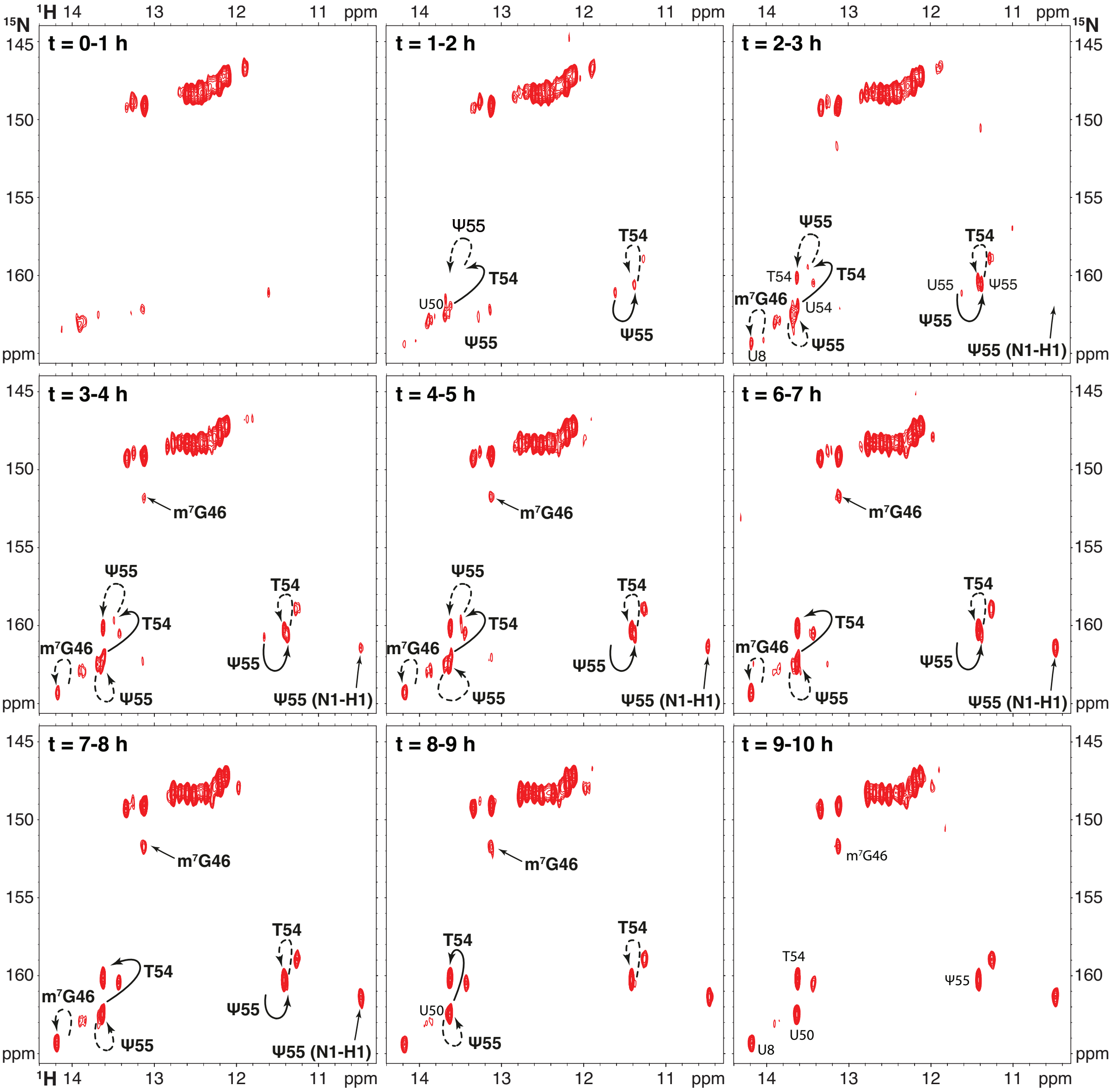

Supplementary Figure S12: Time-resolved NMR monitoring of *E. coli* tRNA^{Phe} maturation in *dusCΔ* cell extracts

(*spectra from top to bottom right*) Imino (^{1}H,^{15}N) correlation spectra of a ^{15}N-labelled tRNA^{Phe} measured in a time-resolved fashion during a continuous incubation at 30°C in *E. coli* extract from a *dusCΔ* strain over 10 h. DusC is responsible for D16 formation. Each NMR spectrum measurement corresponds to a 1 hour time period, as indicated on the top-left corner of each spectrum. Detected modifications are reported with continuous line arrows for direct effects, or dashed arrows for indirect effects.

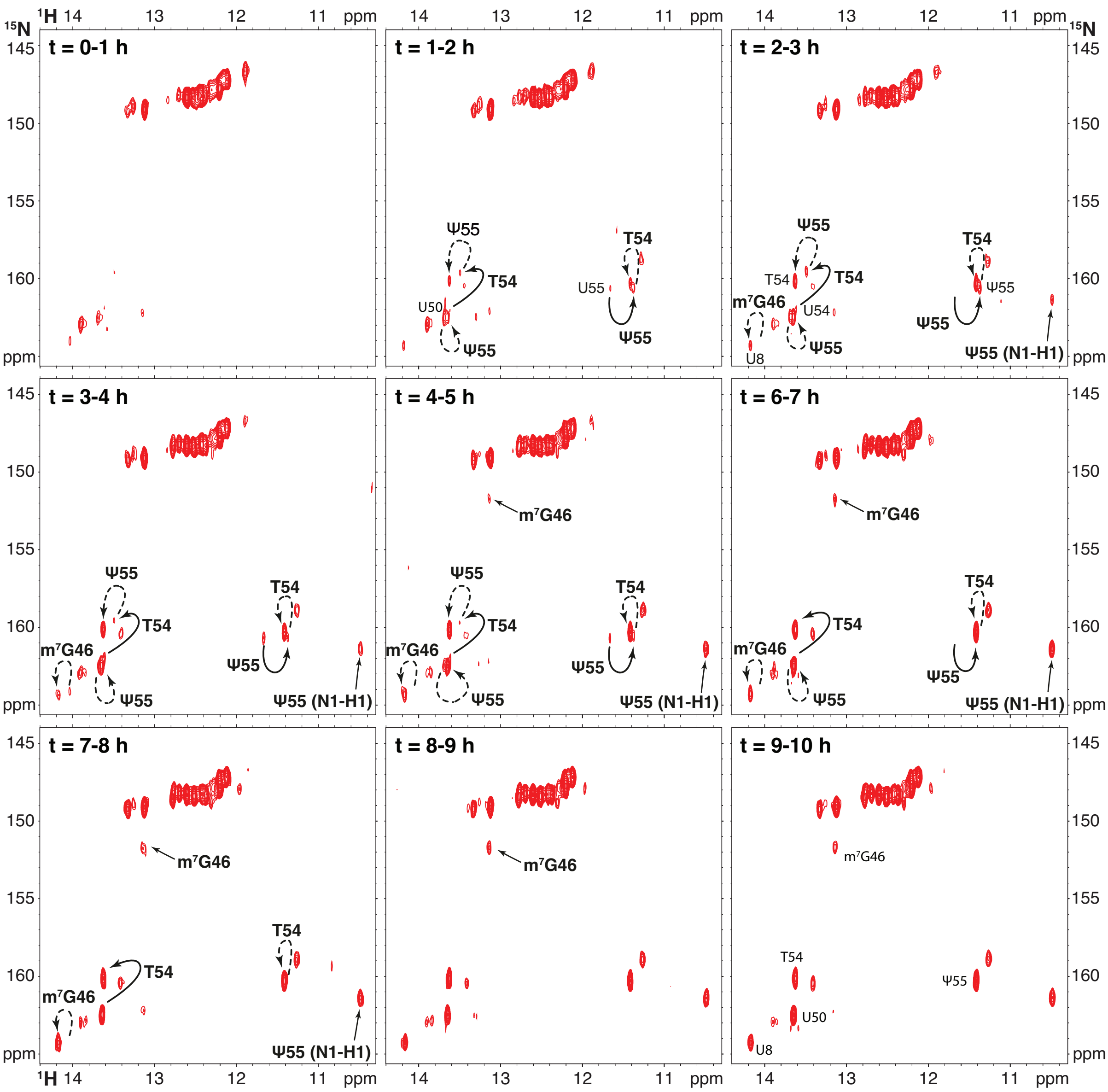

Supplementary Figure S13: Time-resolved NMR monitoring of *E. coli* tRNA^{Phe} maturation in *dusAΔ* cell extracts

(*spectra from top to bottom right*) Imino (^{1}H,^{15}N) correlation spectra of a ^{15}N-labelled tRNA^{Phe} measured in a time-resolved fashion during a continuous incubation at 30°C in *E. coli* extract from a *dusAΔ* strain over 10 h. DusA is responsible for D20 formation in this tRNA. Each NMR spectrum measurement corresponds to a 1 hour time period, as indicated on the top-left corner of each spectrum. Detected modifications are reported with continuous line arrows for direct effects, or dashed arrows for indirect effects.

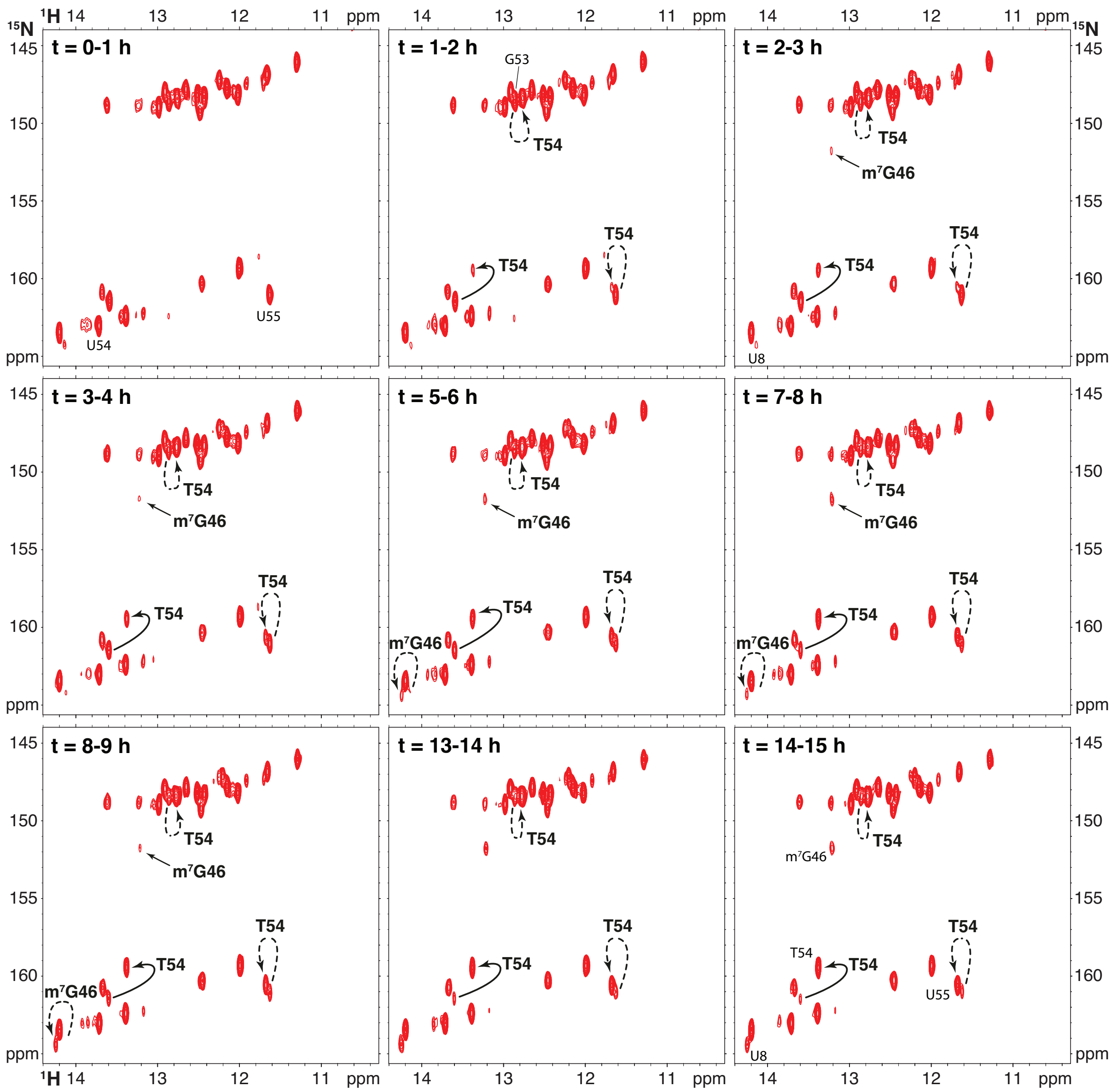

Supplementary Figure S14: Time-resolved NMR monitoring of *E. coli* tRNA^{Val} maturation in *truBΔ* cell extracts

(*spectra from top to bottom right*) Imino (^{1}H,^{15}N) correlation spectra of a ^{15}N-labelled tRNA^{Val} measured in a time-resolved fashion during a continuous incubation at 30°C in *E. coli* extract from a *truBΔ* strain over 15 h. TruB is responsible for Ψ55 formation. Each NMR spectrum measurement corresponds to a 1 hour time period, as indicated on the top-left corner of each spectrum. Detected modifications are reported with continuous line arrows for direct effects, or dashed arrows for indirect effects.

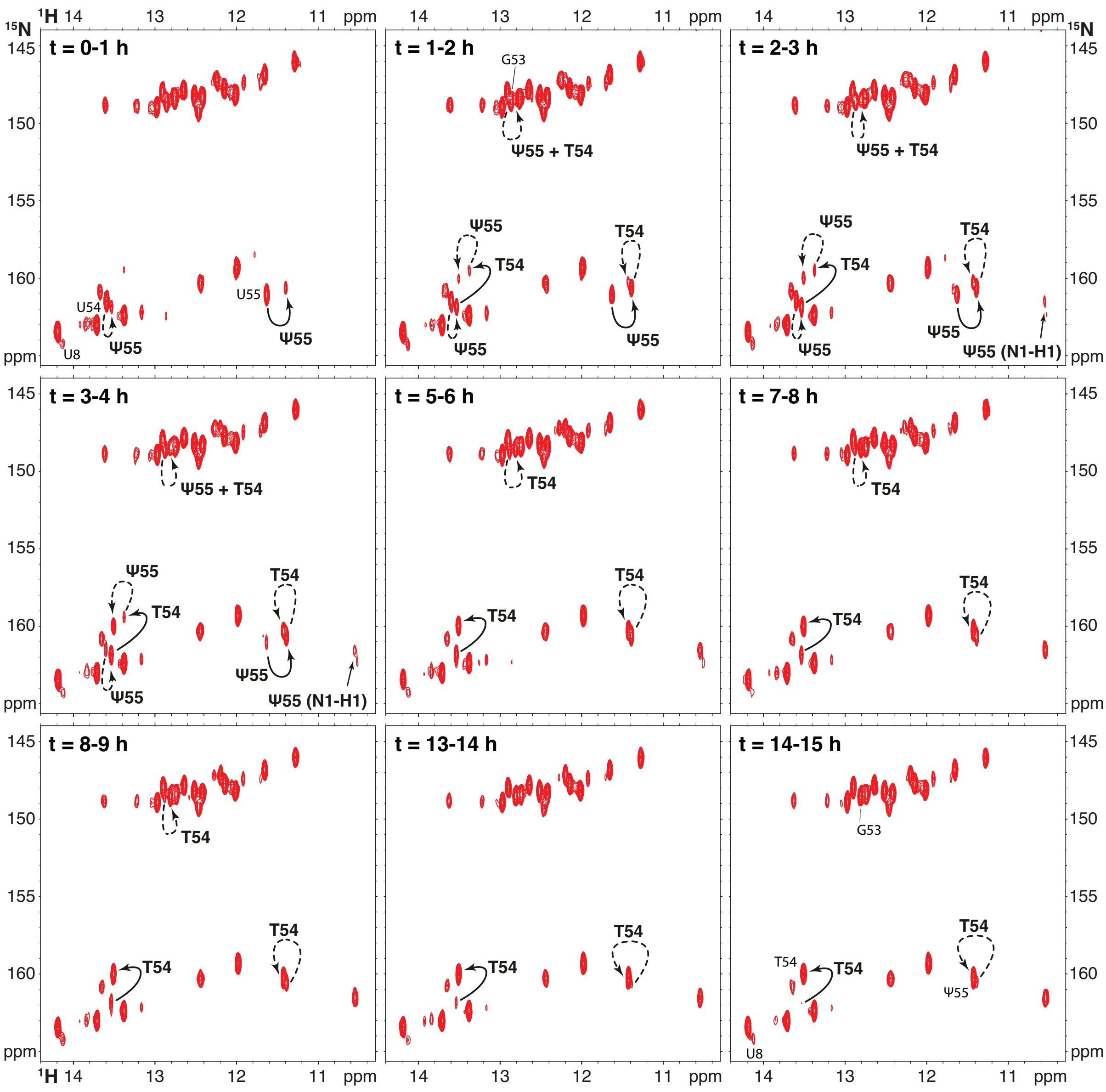

Supplementary Figure S15: Time-resolved NMR monitoring of *E. coli* tRNA^{Val} maturation in *trmBΔ* cell extracts

(*spectra from top to bottom right*) Imino (^{1}H,^{15}N) correlation spectra of a ^{15}N-labelled tRNA^{Val} measured in a time-resolved fashion during a continuous incubation at 30°C in *E. coli* extract from a *trmBΔ* strain over 15 h. TrmB is responsible for m^{7}G46 formation. Each NMR spectrum measurement corresponds to a 1 hour time period, as indicated on the top-left corner of each spectrum. Detected modifications are reported with continuous line arrows for direct effects, or dashed arrows for indirect effects.

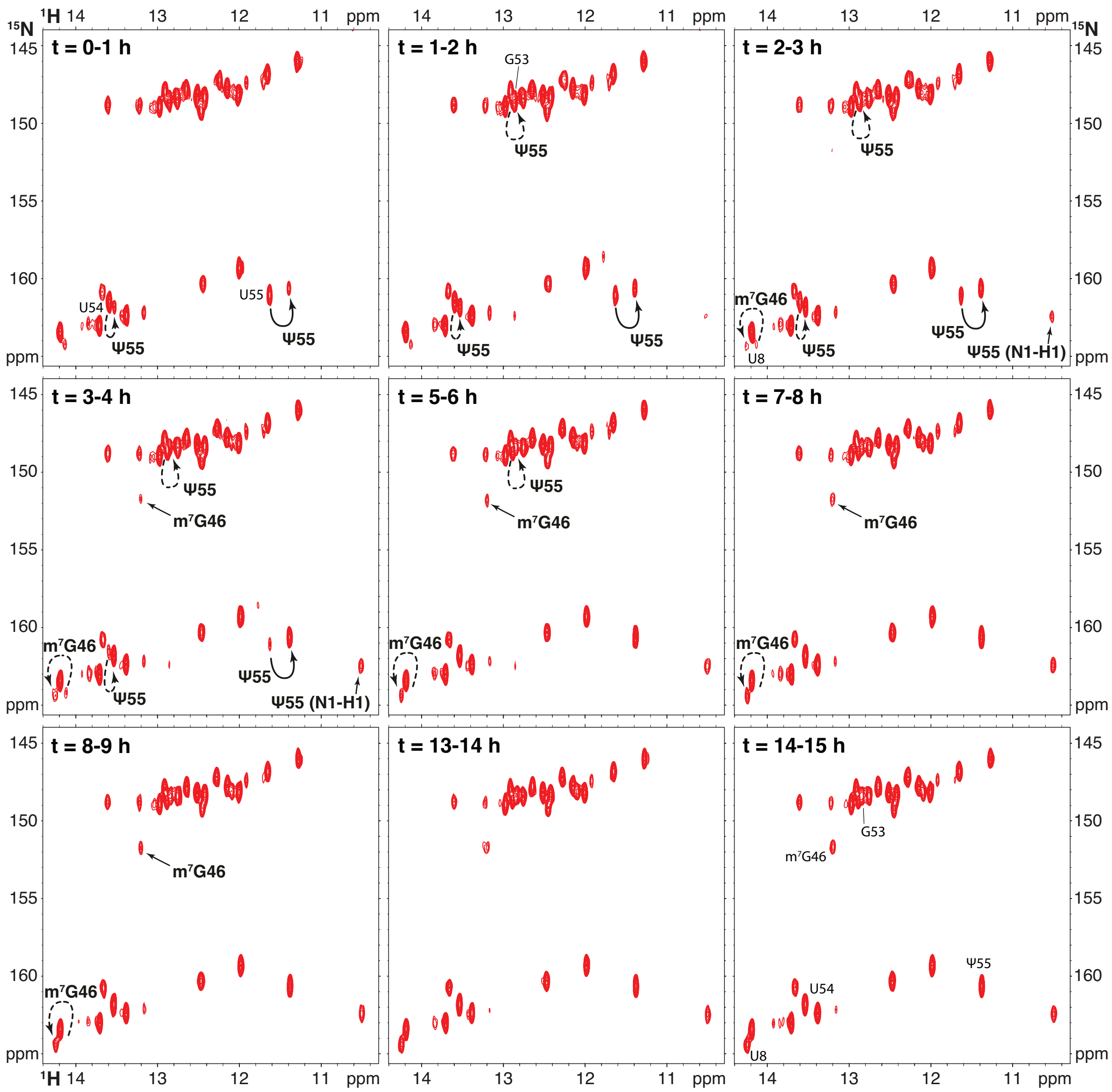

Supplementary Figure S16: Time-resolved NMR monitoring of *E. coli* tRNA^{Val} maturation in *trmAΔ* cell extracts

(*spectra from top to bottom right*) Imino (^{1}H,^{15}N) correlation spectra of a ^{15}N-labelled tRNA^{Val} measured in a time-resolved fashion during a continuous incubation at 30°C in *E. coli* extract from a *trmAΔ* strain over 15 h. TrmA is responsible for T54 formation. Each NMR spectrum measurement corresponds to a 1 hour time period, as indicated on the top-left corner of each spectrum. Detected modifications are reported with continuous line arrows for direct effects, or dashed arrows for indirect effects.

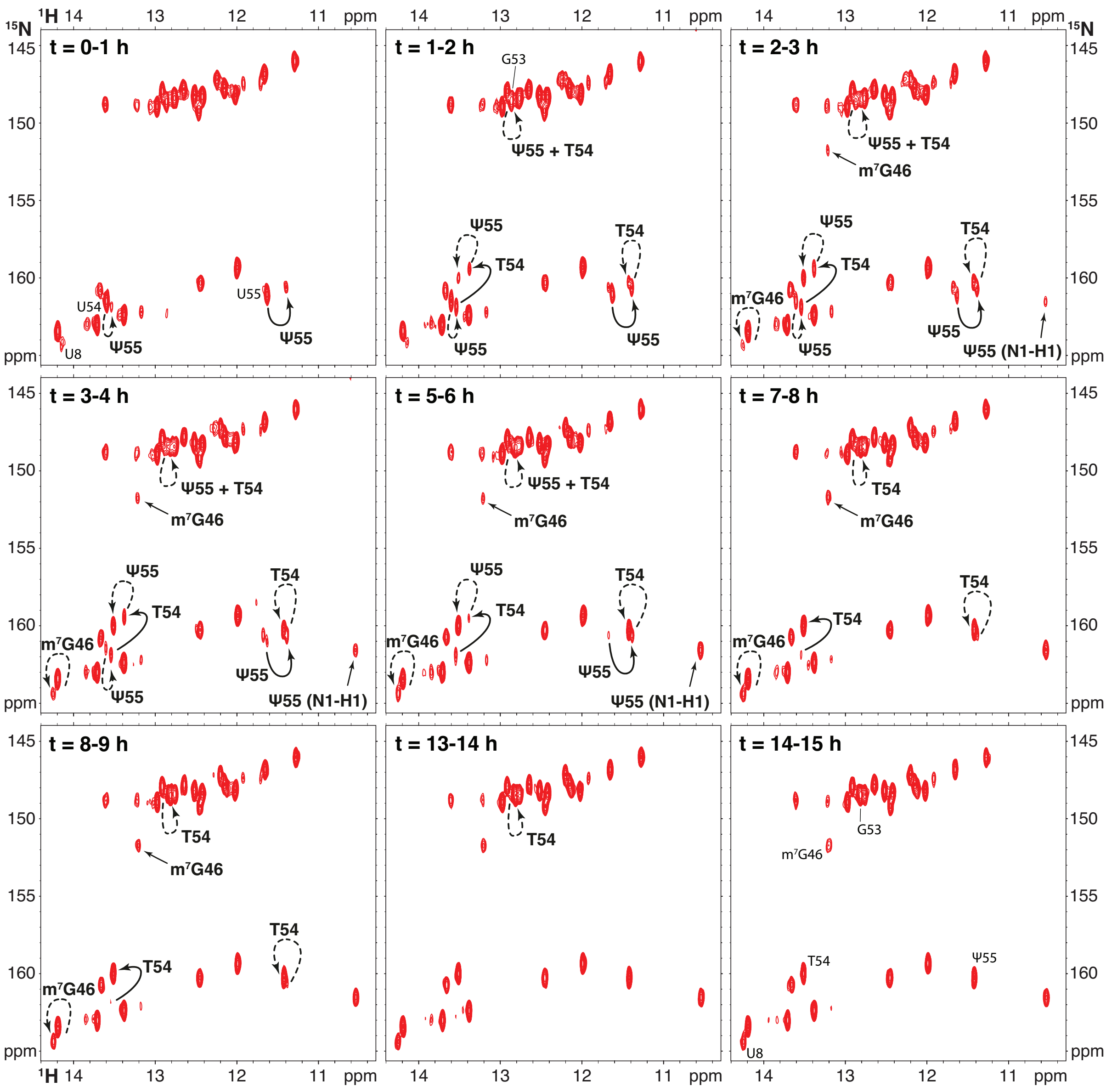

Supplementary Figure S17: Time-resolved NMR monitoring of *E. coli* tRNA^{Val} maturation in *thiIΔ* cell extracts

(*spectra from top to bottom right*) Imino (^{1}H,^{15}N) correlation spectra of a ^{15}N-labelled tRNA^{Val} measured in a time-resolved fashion during a continuous incubation at 30°C in *E. coli* extract from a *thiIΔ* strain over 15 h. ThiI is responsible for s^{4}U8 formation. Each NMR spectrum measurement corresponds to a 1 hour time period, as indicated on the top-left corner of each spectrum. Detected modifications are reported with continuous line arrows for direct effects, or dashed arrows for indirect effects.

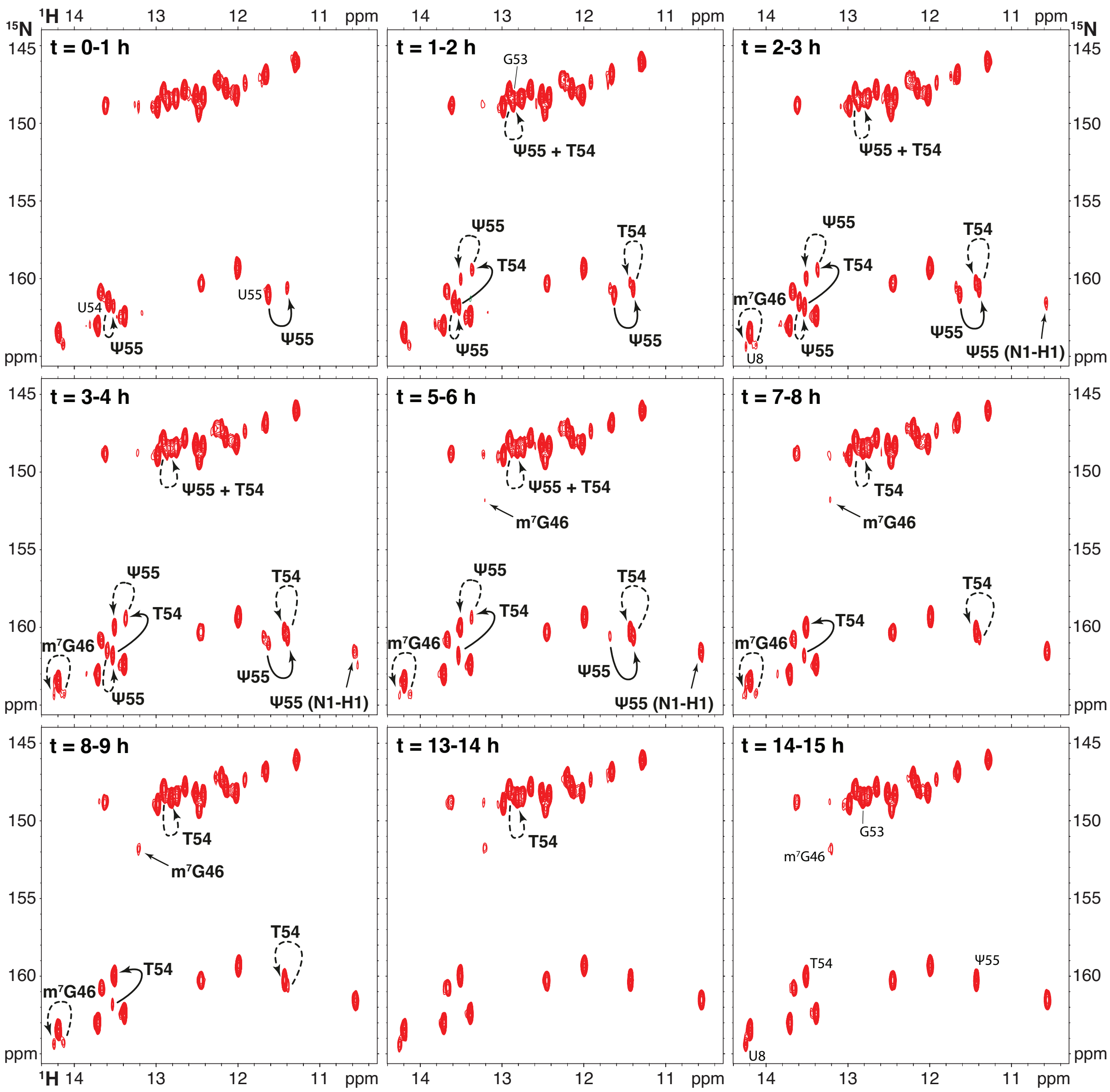

Supplementary Figure S18: Time-resolved NMR monitoring of *E. coli* tRNA^{Val} maturation in *dusBΔ* cell extracts

(*spectra from top to bottom right*) Imino (^{1}H,^{15}N) correlation spectra of a ^{15}N-labelled tRNA^{Val} measured in a time-resolved fashion during a continuous incubation at 30°C in *E. coli* extract from a *dusBΔ* strain over 15 h. DusB is responsible for D17 formation. Each NMR spectrum measurement corresponds to a 1 hour time period, as indicated on the top-left corner of each spectrum. Detected modifications are reported with continuous line arrows for direct effects, or dashed arrows for indirect effects.

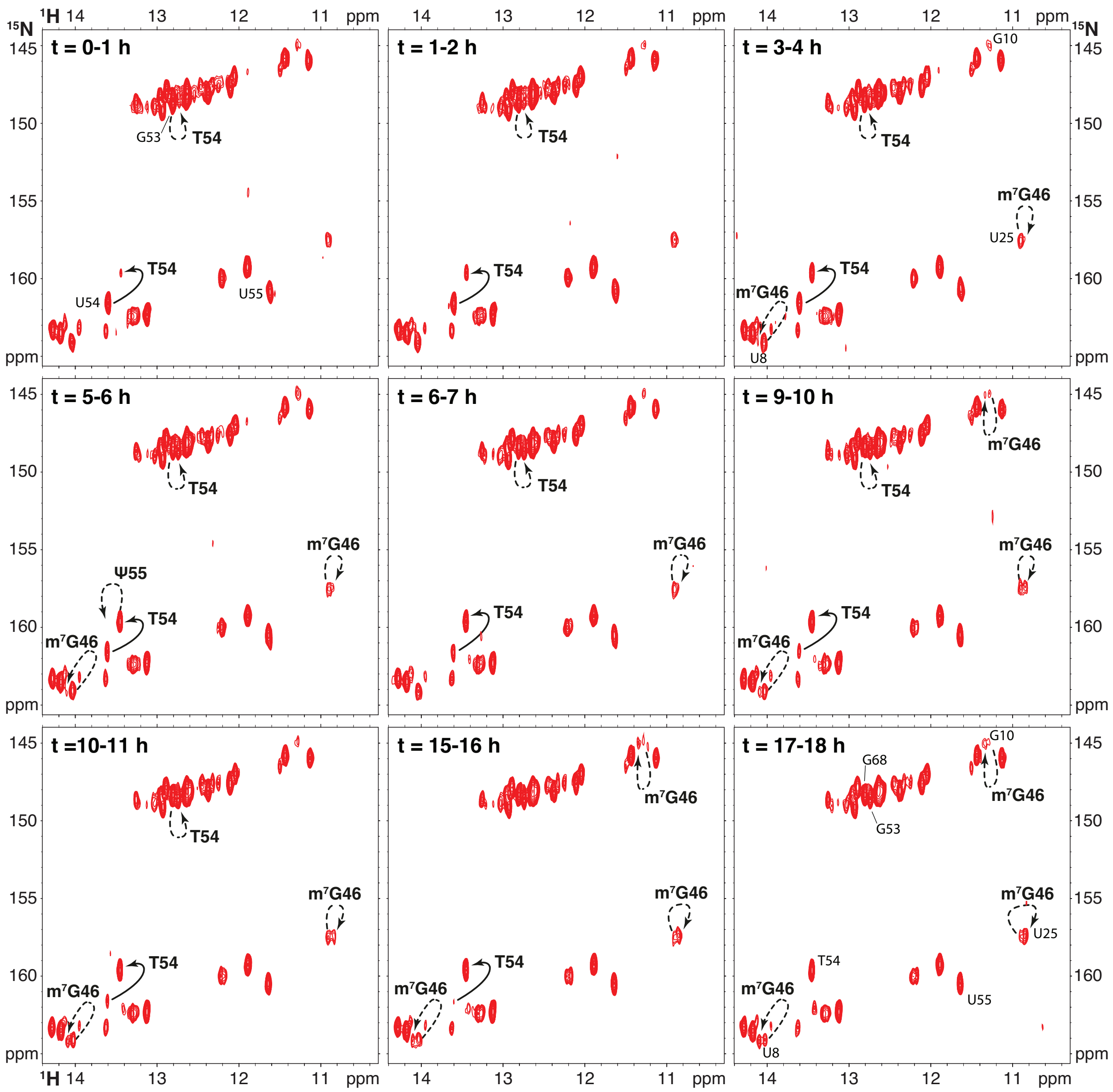

Supplementary Figure S19: Time-resolved NMR monitoring of *E. coli* tRNA^{Asp} maturation in *truBΔ* cell extracts

(*spectra from top to bottom right*) Imino (^{1}H,^{15}N) correlation spectra of a ^{15}N-labelled tRNA^{Asp} measured in a time-resolved fashion during a continuous incubation at 30°C in *E. coli* extract from a *truBΔ* strain over 18 h. TruB is responsible for Ψ55 formation. Each NMR spectrum measurement corresponds to a 1 hour time period, as indicated on the top-left corner of each spectrum. Detected modifications are reported with continuous line arrows for direct effects, or dashed arrows for indirect effects.

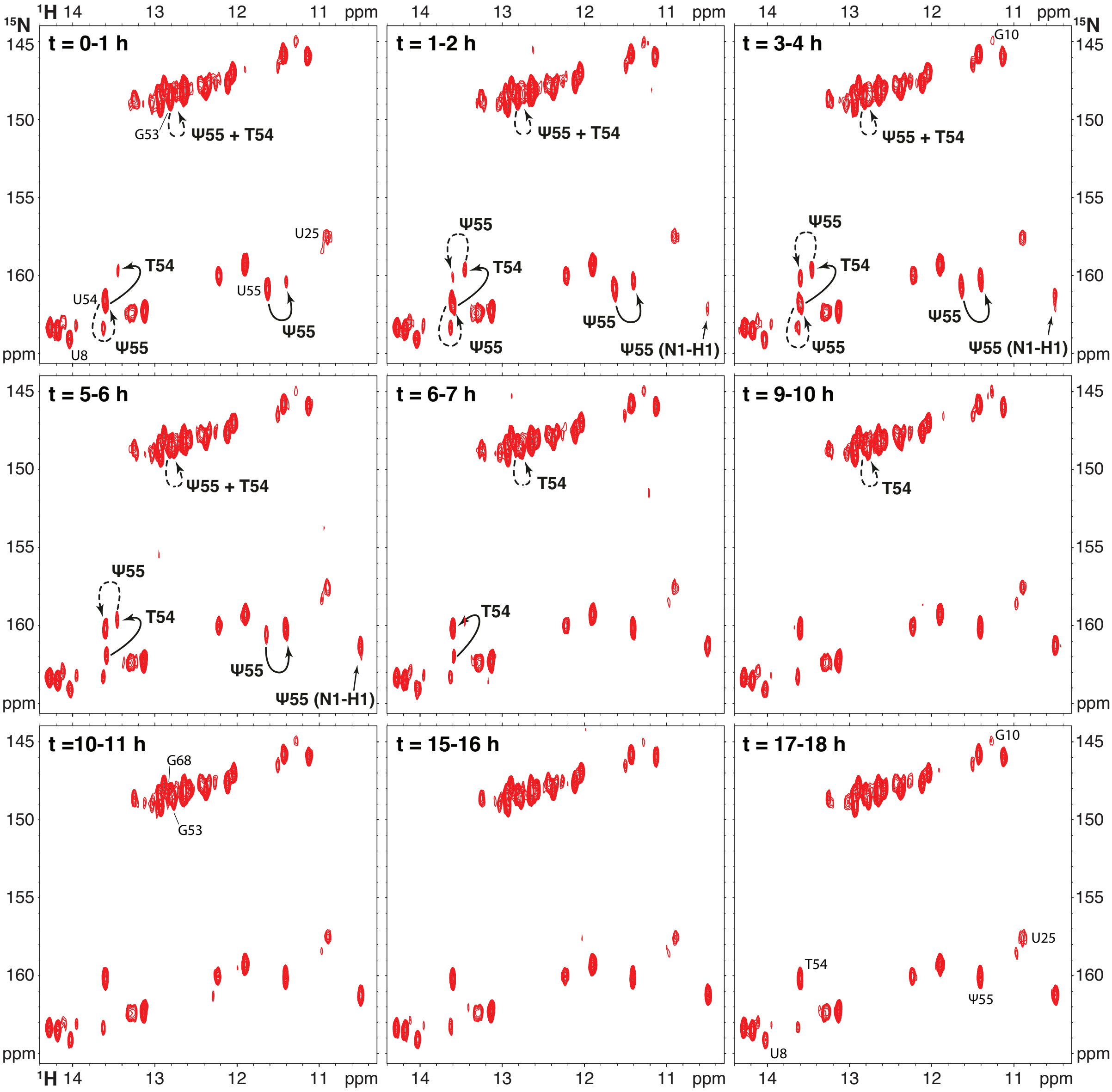

Supplementary Figure S20: Time-resolved NMR monitoring of *E. coli* tRNA^{Asp} maturation in *trmBΔ* cell extracts

(*spectra from top to bottom right*) Imino (^{1}H,^{15}N) correlation spectra of a ^{15}N-labelled tRNA^{Asp} measured in a time-resolved fashion during a continuous incubation at 30°C in *E. coli* extract from a *trmBΔ* strain over 18 h. TrmB is responsible for m^{7}G46 formation. Each NMR spectrum measurement corresponds to a 1 hour time period, as indicated on the top-left corner of each spectrum. Detected modifications are reported with continuous line arrows for direct effects, or dashed arrows for indirect effects.

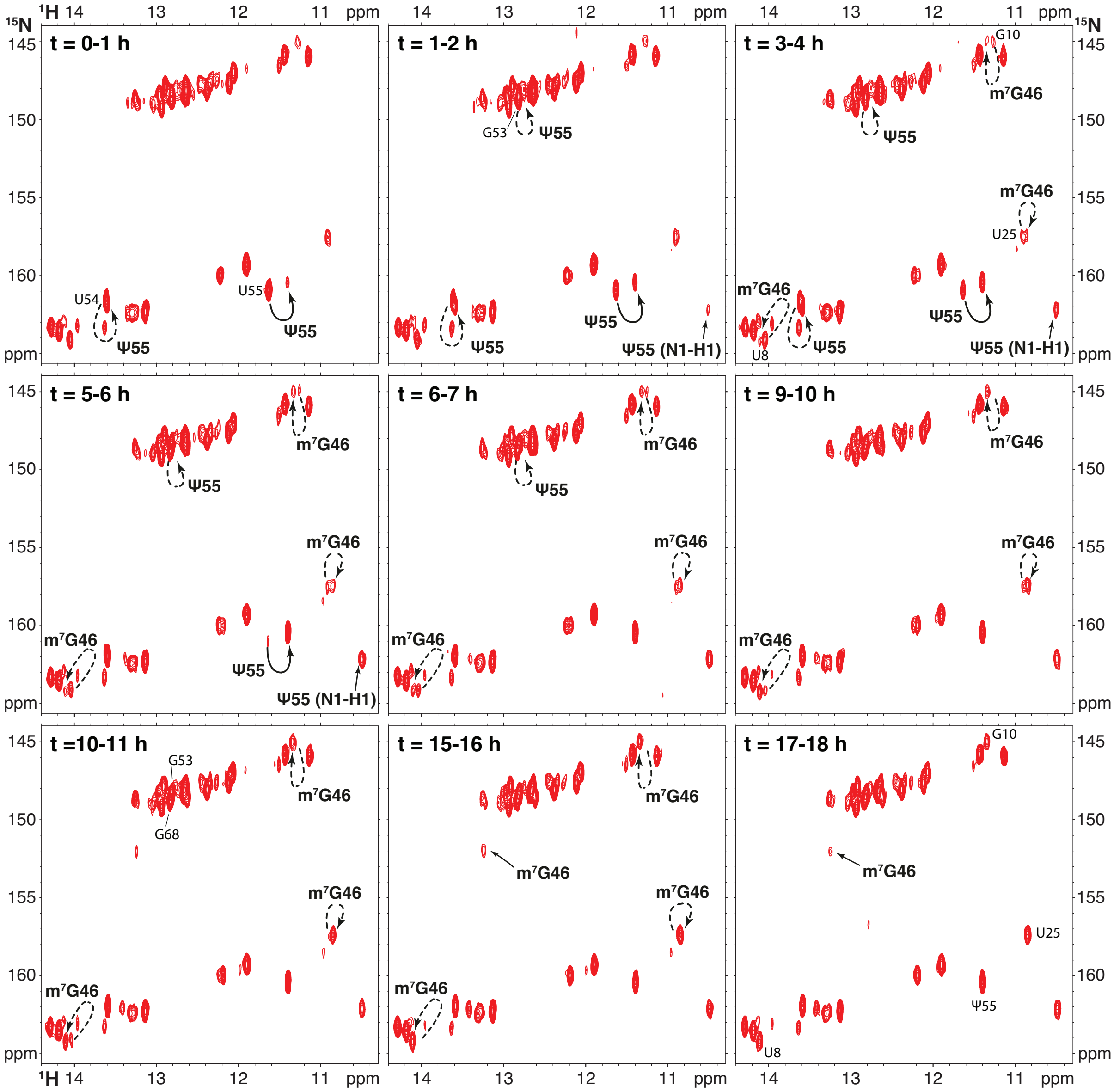

Supplementary Figure S21: Time-resolved NMR monitoring of *E. coli* tRNA^{Asp} maturation in *trmAΔ* cell extracts

(*spectra from top to bottom right*) Imino (^{1}H,^{15}N) correlation spectra of a ^{15}N-labelled tRNA^{Asp} measured in a time-resolved fashion during a continuous incubation at 30°C in *E. coli* extract from a *trmAΔ* strain over 18 h. TrmA is responsible for T54 formation. Each NMR spectrum measurement corresponds to a 1 hour time period, as indicated on the top-left corner of each spectrum. Detected modifications are reported with continuous line arrows for direct effects, or dashed arrows for indirect effects.

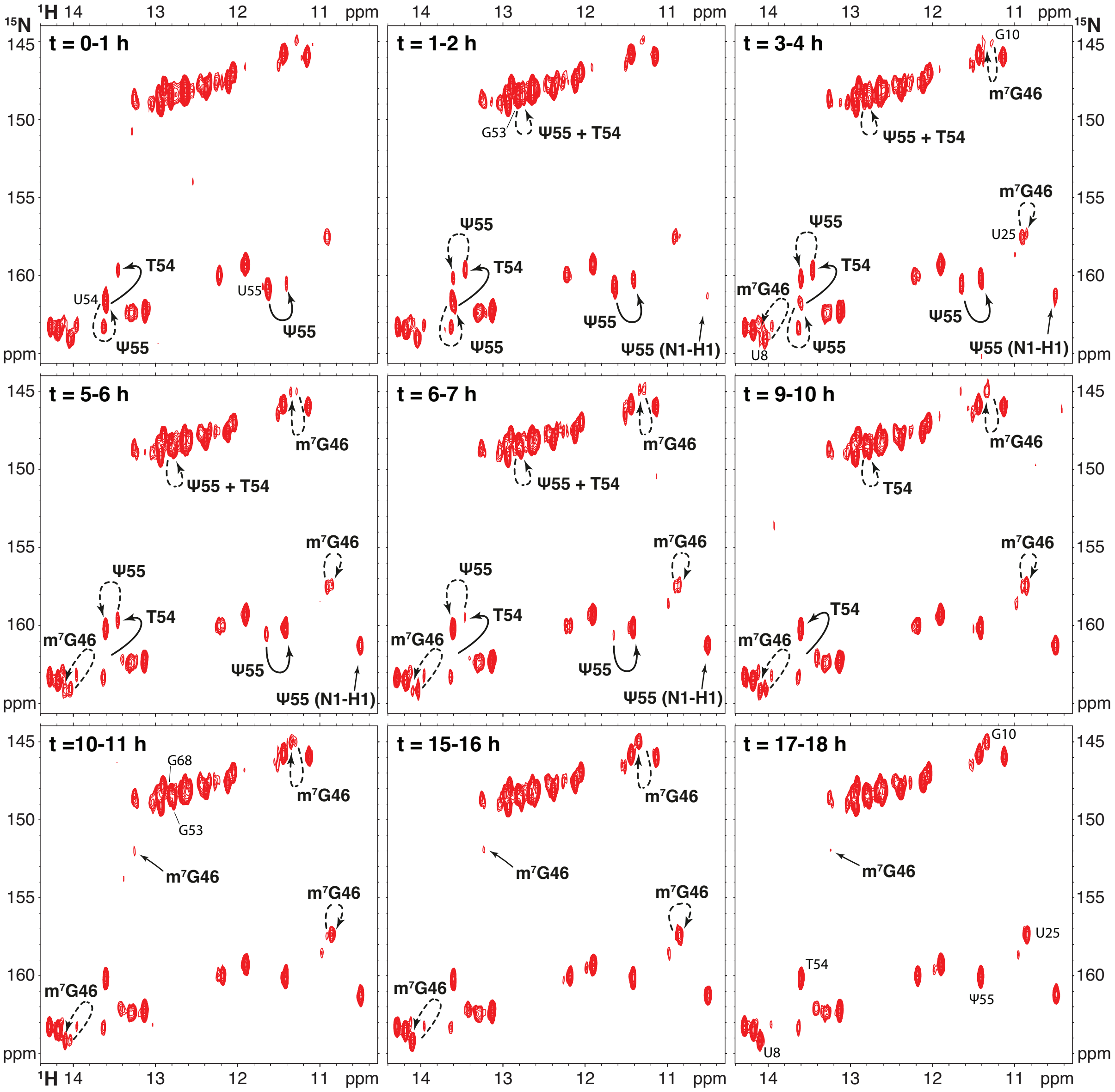

Supplementary Figure S22: Time-resolved NMR monitoring of *E. coli* tRNA^{Asp} maturation in *thiIΔ* cell extracts

(*spectra from top to bottom right*) Imino (^{1}H,^{15}N) correlation spectra of a ^{15}N-labelled tRNA^{Asp} measured in a time-resolved fashion during a continuous incubation at 30°C in *E. coli* extract from a *thiIΔ* strain over 18 h. ThiI is responsible for s^{4}U8 formation. Each NMR spectrum measurement corresponds to a 1 hour time period, as indicated on the top-left corner of each spectrum. Detected modifications are reported with continuous line arrows for direct effects, or dashed arrows for indirect effects.

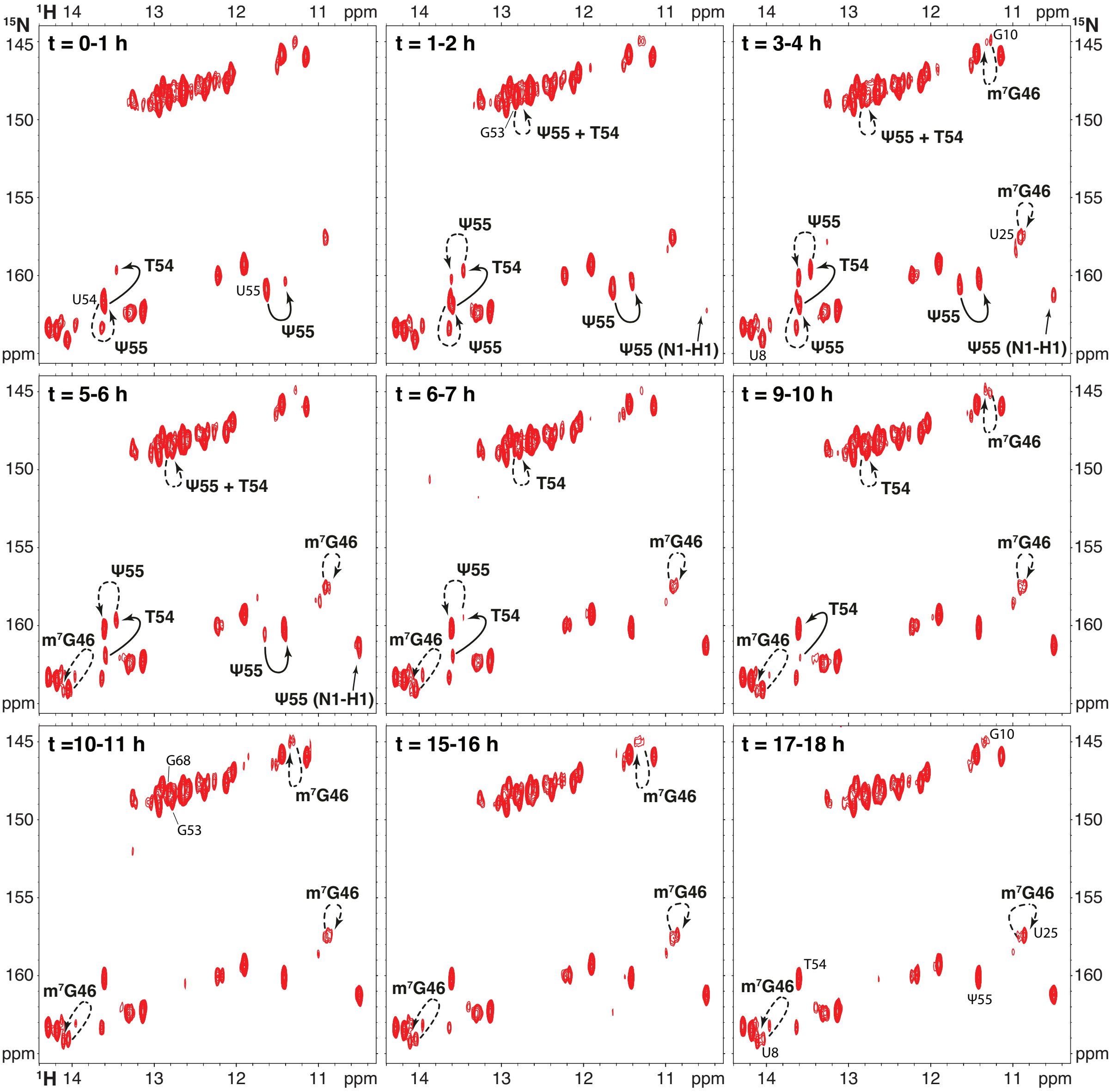

Supplementary Figure S23: Time-resolved NMR monitoring of *E. coli* tRNA^{Asp} maturation in *dusAΔ* cell extracts

(*spectra from top to bottom right*) Imino (^{1}H,^{15}N) correlation spectra of a ^{15}N-labelled tRNA^{Asp} measured in a time-resolved fashion during a continuous incubation at 30°C in *E. coli* extract from a *dusAΔ* strain over 18 h. DusA is responsible for D20 and D20a formation in this tRNA. Each NMR spectrum measurement corresponds to a 1 hour time period, as indicated on the top-left corner of each spectrum. Detected modifications are reported with continuous line arrows for direct effects, or dashed arrows for indirect effects.

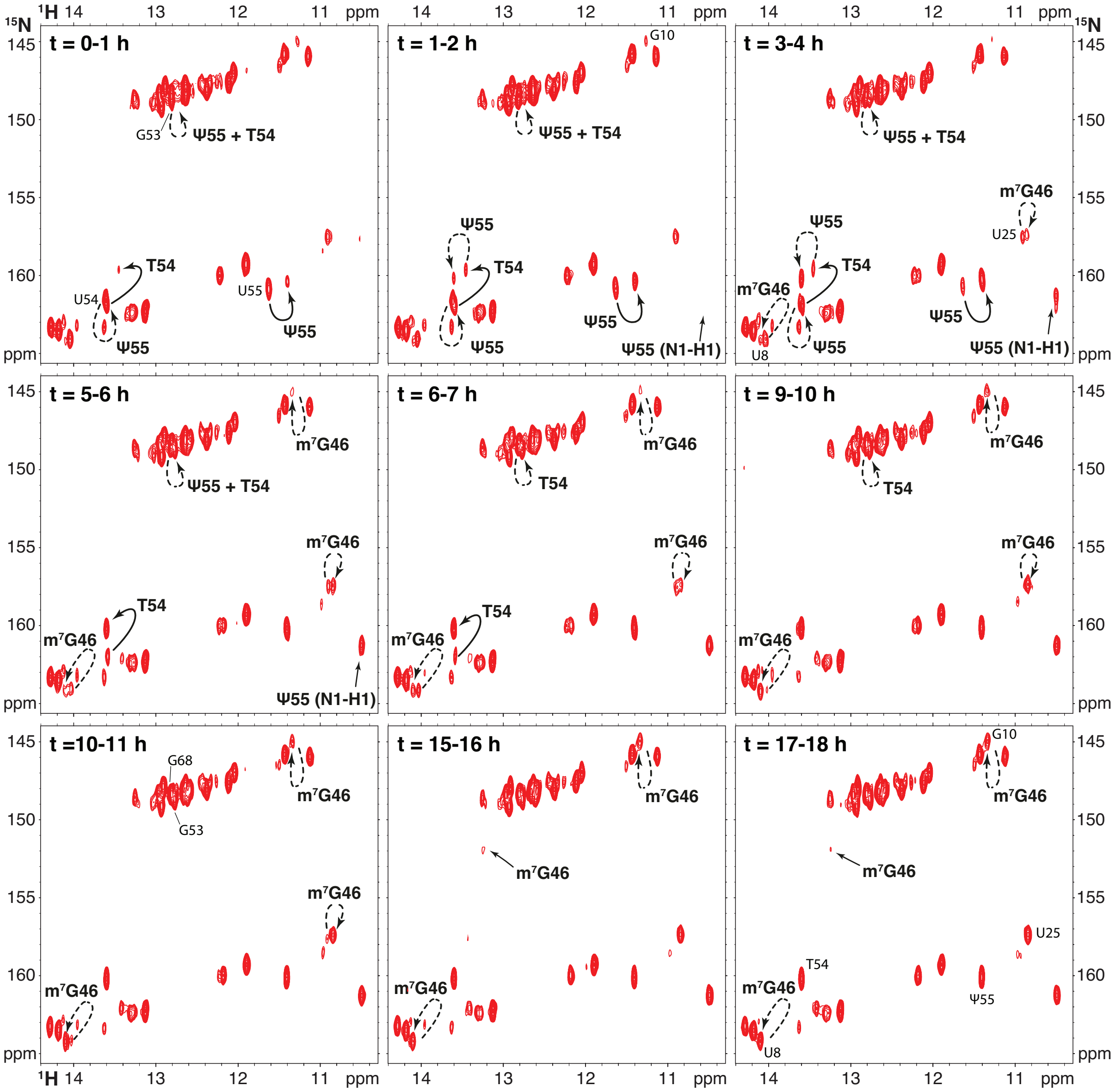

Supplementary Figure S24: Time-resolved NMR monitoring of *E. coli* tRNA^{Asp} maturation in *dusCΔ* cell extracts

(*spectra from top to bottom right*) Imino (^{1}H,^{15}N) correlation spectra of a ^{15}N-labelled tRNA^{Asp} measured in a time-resolved fashion during a continuous incubation at 30°C in *E. coli* extract from a *dusCΔ* strain over 18 h. DusC is responsible for D16 formation. Each NMR spectrum measurement corresponds to a 1 hour time period, as indicated on the top-left corner of each spectrum. Detected modifications are reported with continuous line arrows for direct effects, or dashed arrows for indirect effects.

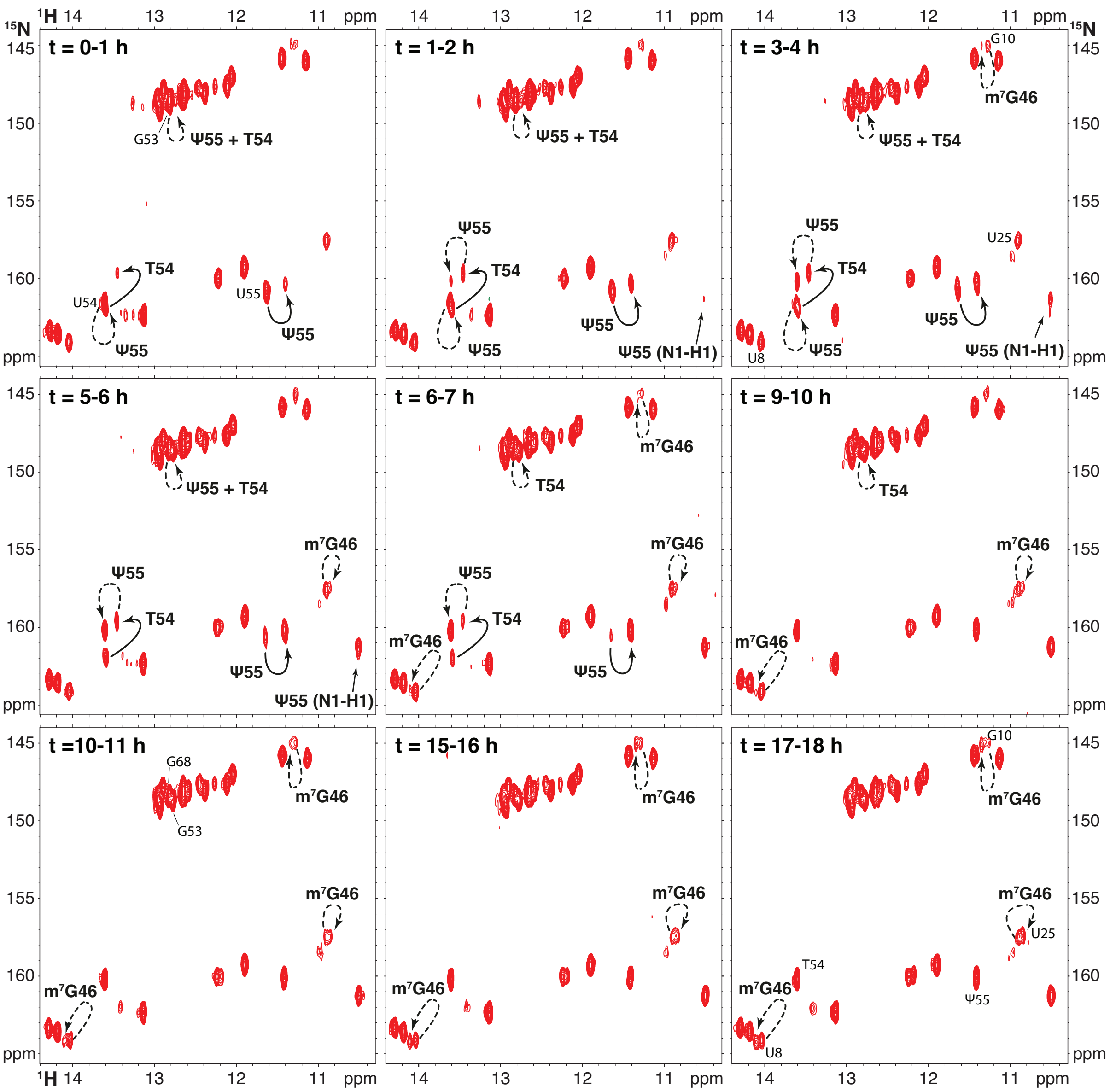

Supplementary Figure S25: Time-resolved NMR monitoring of *E. coli* tRNA^{Asp} maturation in *truCΔ* cell extracts

(*spectra from top to bottom right*) Imino (^{1}H,^{15}N) correlation spectra of a ^{15}N-labelled tRNA^{Asp} measured in a time-resolved fashion during a continuous incubation at 30°C in *E. coli* extract from a *truCΔ* strain over 18 h. TruC is responsible for Ψ65 formation. Each NMR spectrum measurement corresponds to a 1 hour time period, as indicated on the top-left corner of each spectrum. Detected modifications are reported with continuous line arrows for direct effects, or dashed arrows for indirect effects.

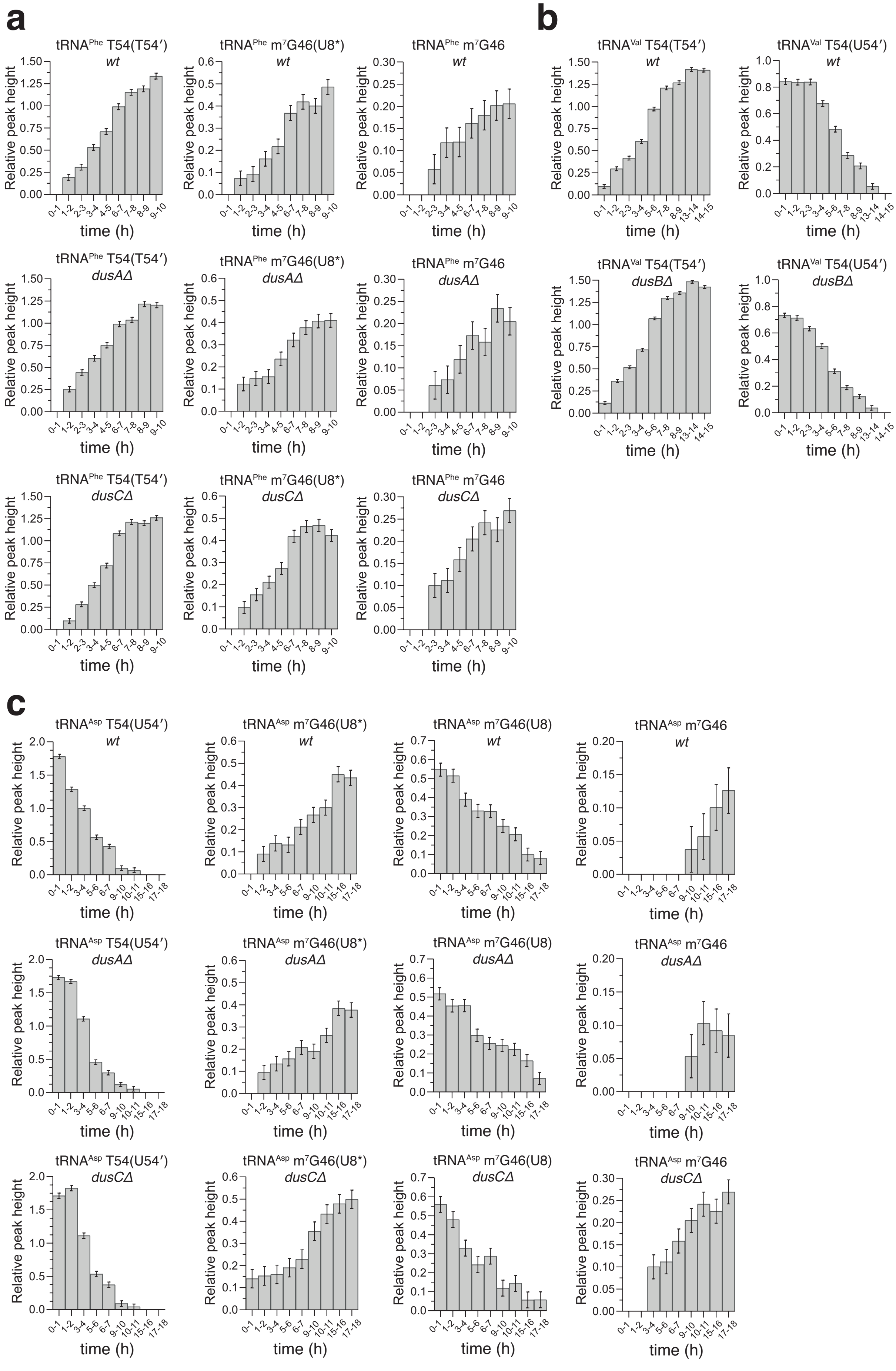
a
tRNAPhe T54(T54′)
wt
tRNAPhe m7G46(U8*)
wt
tRNAPhe m7G46
wt
tRNAPhe T54(T54′)
dusAΔ
tRNAPhe m7G46(U8*)
dusAΔ
tRNAPhe m7G46
dusAΔ
tRNAPhe T54(T54′)
dusCΔ
tRNAPhe m7G46(U8*)
dusCΔ
tRNAPhe m7G46
dusCΔ
b
tRNAVal T54(T54′)
wt
tRNAVal T54(U54′)
wt
tRNAVal T54(T54′)
dusBΔ
tRNAVal T54(U54′)
dusBΔ
c
tRNAAsp T54(U54′)
wt
tRNAAsp m7G46(U8*)
wt
tRNAAsp m7G46(U8)
wt
tRNAAsp m7G46
wt
tRNAAsp T54(U54′)
dusAΔ
tRNAAsp m7G46(U8*)
dusAΔ
tRNAAsp m7G46(U8)
dusAΔ
tRNAAsp m7G46
dusAΔ
tRNAAsp T54(U54′)
dusCΔ
tRNAAsp m7G46(U8*)
dusCΔ
tRNAAsp m7G46(U8)
dusCΔ
tRNAAsp m7G46
dusCΔ
Relative peak height
time (h)

Supplementary Figure S26: Analysis of time-resolved NMR maturation of *E. coli* tRNA^{Phe}, tRNA^{Val}, and tRNA^{Asp} in *wild-type*, and Dus deletion strains (i.e. *dusAΔ*, *dusBΔ* and *dusCΔ*) used as control strains.

(**a**) Time-resolved evolution of the relative peak height of NMR signals reporting on the formation of the T54 and m^{7}G46 modifications in tRNA^{Phe}, monitored in wild-type *(wt)*, *dusAΔ* and *dusCΔ* extracts. (**b**) Time-resolved evolution of the relative peak height of NMR signals reporting on T54 formation in tRNA^{Val}, monitored in *wt* and *dusBΔ* cell extracts. (**c**) Time-resolved evolution of the relative peak height of NMR signals reporting on T54 and m^{7}G46 formation in tRNA^{Asp}, monitored in *wt*, *dusAΔ* and *dusCΔ* extracts. In all series, the relative peak height value for T54′/U54′ includes the contribution of the T54/U54 peak (tRNA with unmodified U55) and the shifted T54*/U54* peak (tRNA with modified Ψ55). Error bars represent the reported uncertainty in peak heights, calculated as ±2 × the normalized mean RMS of the noise, thereby approximating a 95% confidence interval (CI 95%; see Experimental Procedures).
